# Injectable Hierarchical Bioactive Hydrogels with Fibroblast Growth Factor 21/Edaravone/Caffeic Acid Asynchronous Delivery for Treating Parkinson's Disease

**DOI:** 10.1002/advs.202412020

**Published:** 2024-12-04

**Authors:** Junpeng Xu, Peng Dai, Chen Zhang, Na Dong, Caiyan Li, Chonghui Tang, Zhihao Jin, Shih‐Ho Lin, Luyang Ye, Tianmiao Sun, Yukai Jin, Fenzan Wu, Lihua Luo, Ping Wu, Shengcun Li, Xiaokun Li, Shan‐hui Hsu, Dawei Jiang, Zhouguang Wang

**Affiliations:** ^1^ Affiliated Cixi Hospital Wenzhou Medical University Ningbo Zhejiang 315300 China; ^2^ Oujiang Laboratory (Zhejiang Lab for Regenerative Medicine, Vision and Brain Health) School of Pharmaceutical Science Wenzhou Medical University Wenzhou Zhejiang 325000 China; ^3^ State Key Laboratory of Macromolecular Drugs and Large‐scale Preparation School of Pharmaceutical Science Wenzhou Medical University Wenzhou Zhejiang 325000 China; ^4^ School and Hospital of Stomatology Wenzhou Medical University Wenzhou Zhejiang 324025 China; ^5^ Institute of Polymer Science and Engineering National Taiwan University Taipei Taiwan 106319 Republic of China; ^6^ Rehabilitation Medicine Center The Second Affiliated Hospital and Yuying Children's Hospital of Wenzhou Medical University Wenzhou Zhejiang 325000 China; ^7^ Institute of Cellular and System Medicine National Health Research Institutes Miaoli Taiwan 350401 Republic of China

**Keywords:** asynchronous release, bioactive hydrogel, fibroblast growth factor, Parkinson's disease, triple‐drug delivery system

## Abstract

Parkinson's disease (PD) is one of the most common long‐term neurodegenerative disorders, with multiple comorbid psychiatric and behavioral abnormalities. The combination of clinical drugs targeting different symptoms with smart hydrogels to achieve asynchronous releases is highly translational and challenging. Here, a hierarchical bioactive hydrogel (OACDP) is designed with asynchronous release based on PD pathology. The hydrogel with caffeic acid‐grafted polymer main chain is crosslinked using a micellar nanocrosslinker, with sufficient modulus (≈167 Pa), antioxidant activity (> 50%), injectability (30‐gauge syringe needle), and shape‐adaptability. Each of the three drugs (caffeic acid, fibroblast growth factor 21, and Edaravone) is separately engaged in different micro‐ or nanostructures of the hydrogel and released with asynchronous kinetics of first‐order release, zero‐order release, or matching Korsmeyer–Peppas model. The triple‐loaded hydrogel is injected into the brains of PD rats, showing behavioral improvement. Histological analysis revealed that the triple‐loaded OACDP hydrogels are effective in achieving immediate neuroprotection, i.e., reduction the loss of tyrosine hydroxylase in substantia nigra compacta and striatum (retained ≈10‐fold versus control), decreasing oxidative stress, reducing astrocyte and microglia activation, and stimulating the AMPK/PGC‐1α axis to regulate the mitochondrial function, providing a multi‐dimensional PD therapy. The asynchronous release of OACDP hydrogel provides a new conception for PD treatment and other neurodegenerative diseases.

## Graphical abstract

A hierarchical bioactive hydrogel (OACDP) targeting Parkinson's disease is developed, enabling the asynchronous release of three drugs (caffeic acid, fibroblast growth factor 21, and Edaravone). The hydrogel possesses adequate antioxidant properties and tiny needle injectability. Post‐injection in Parkinsonian rats, triple‐loaded OACDP hydrogel results in significant behavioral improvements, offering promising multidimensional neuroprotection and therapeutic potential for neurodegenerative diseases.

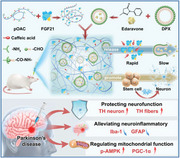

## 1 Introduction

Parkinson's disease (PD), being the second most common neurodegenerative disease, is a chronic progressive age‐related disorder.^[^ ^1^ ^]^ PD is characterized by cellular loss and functional degeneration of nigrostriatal dopaminergic neurons that project to the striatum.^[^ ^2^ ^]^ Major factors contributing to the aggressive course of PD are oxidative stress‐stimulated neuroinflammation and mitochondrial dysfunction. Oxidative stress triggers a feed‐forward loop of neuroinflammation and neuronal apoptosis.^[^ ^3^ ^]^ In addition, mitochondrial function is aberrated by the accumulation of reactive oxygen species (ROS), resulting in the aggregation of α‐synuclein, which promotes neuroinflammation and exacerbates the course of PD.^[^ ^4^ ^]^ Albeit no cure for PD exists at present, clinical methods are available to help patients alleviate their symptoms of abnormal motor function via deep brain stimulation (DBS) or medication.^[^ ^5^ ^]^ However, DBS carries a high cost and the risk of intracranial hemorrhage and infection associated with prolonged retention of the electrodes, along with side effects such as dizziness and nausea after treatment. Although the early stage of PD can be managed by monotherapy, many PD patients are suffering from the intermediate to advanced stages of PD, which are complicated and accompanied by multiple symptoms. When PD progresses to the advanced stage, oral drug therapy is highly affected by the blood‐brain barrier with low therapeutic efficiency and large individual differences in treatment response, resulting in the inevitable need to use drug combinations or new therapeutic strategies.

The disease stage of PD is divided into phases depending on the severity of the symptoms, and each phase requires the administration of medications to address one or more symptoms.^[^ ^6^ ^]^ Meanwhile, the complex symptoms of PD are associated with different pathological changes in the affected brain regions, which need to be modulated from multiple pathways.^[^ ^7^ ^]^ Such an asynchronous release cannot be achieved by simply taking multiple drugs orally. Besides, the therapeutic drug system using local administration minimizes the dose of the medication as well as the metabolic catabolism of drugs within the patient's body.^[^ ^8^ ^]^ This new treatment concept can be realized by minimally invasive injection using the idea of precision medicine.^[^ ^9^ ^]^ Through targeted injection of biocompatible dual‐ or multiple‐loaded smart carriers with asynchronous release, a very small amount of drug can be used to achieve the purpose of treating multiple symptoms with one single administration. However, such a strategy requires the development of novel smart carriers, and among smart carriers, hydrogels are competitive candidates as biomaterials commonly used for minimally invasive injectable delivery.

Hydrogel has a hydrophilic 3D polymer network structure with a high water content that can mimic the extracellular matrix.^[^ ^10^ ^]^ Self‐healing hydrogel serving as a smart carrier is the development trend of the new generation of biomedical hydrogels.^[^ ^11^ ^]^ Self‐healing hydrogels, inspired by the human body's self‐healing phenomenon, are a class of smart hydrogels with the inherent ability to repair damage spontaneously without external intervention, often featuring injectable and self‐adapting properties.^[^ ^12^ ^]^ The crosslinking mechanisms of self‐healing hydrogels are nowadays developed in various ways, among which Schiff base has attracted more attention due to its mild reaction conditions, easy processing, and the feature of mimicking the self‐healing mechanism in organisms.^[^ ^10^, ^13^ ^]^ The design of polymeric structures for self‐healing hydrogels incorporates functional groups that are beneficial for disease treatment, such as phenolic hydroxyl groups with ROS‐scavenging properties,^[^ ^14^ ^]^ which enable hydrogel carriers to acquire the ability to assist in the treatment of diseases. A chitosan hydrogel crosslinked with a tannic acid‐modified nanogold crosslinker showed a therapeutic effect on PD with the scavenging ability of ROS and the capability of rescuing inflamed neural stem cells (NSCs).^[^ ^15^ ^]^ However, comparisons of therapeutic efficacy with drug‐loaded hydrogels have not been adequately demonstrated, and drug selection should also be more focused on comparing hydrogel with clinical drugs or bioactive drugs that have been reported to be effective, such as fibroblast growth factor (FGF).^[^ ^16^ ^]^

**Figure 1:**
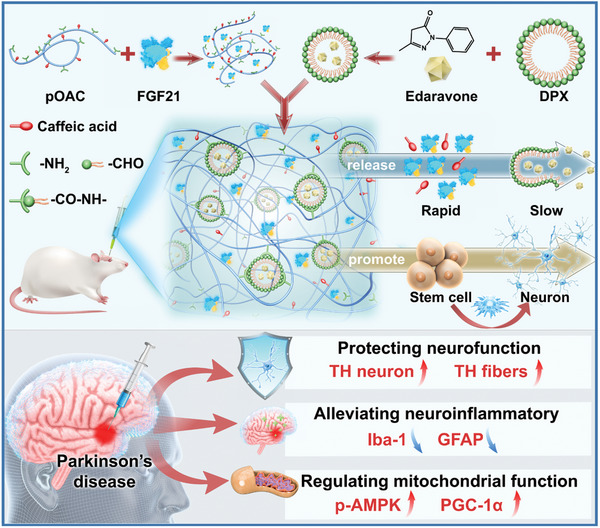
Illustration of the preparation for a bioactive hydrogel with hierarchical structure and the asynchronous release of its three drugs/bioactive substances, including caffeic acid, fibroblast growth factor 21 (FGF21), and Edaravone, as well as its applications for the promotion of neural stem cell differentiation and multi‐dimensional treatment of Parkinson's disease.

FGF is an important family of protein molecules with many subfamilies, and some FGFs like FGF2 have been successfully industrialized and productized.^[^ ^17^ ^]^ Available studies have shown that FGFs have a variety of biological functions, such as promoting cell proliferation, differentiation, and neuroprotection with high potential for clinical translation in the therapeutic area of neurological diseases.^[^ ^17^, ^18^ ^]^ Even though it has been demonstrated that FGF21 could alleviate the degeneration of dopaminergic neurons in PD and improve the motor function of diseased mice,^[^ ^19^ ^]^ the studies integrated dual drugs and hydrogels are very limited and deserve in‐depth research. In the current study, a new antioxidant self‐healing hydrogel (OACDP hydrogel) with unique micro‐ and nanostructure was utilized to simultaneously encapsulate a hydrophilic protein drug (FGF21) and a hydrophobic drug (Edaravone, Eda) as a novel bioactive hydrogel‐based triple‐drug delivery system (TDDS), as shown in **Figure** **1**. Through the asynchronous release characteristics, three drugs are released separately according to the pathogenesis of PD rats in order to achieve immediate neuroprotection, long‐term sustained ROS scavenging, modulation of neuroinflammation, and neural repair. The triple‐loaded OACDP‐treated PD rats showed a significant reduction in behavioral abnormalities and retarded the degeneration/death of dopaminergic neurons and nerve fibers. In vitro cellular assays also confirmed that the triple‐loaded OACDP‐treated group could promote NSC differentiation toward neurons. Asynchronously drug‐releasing OACDPs are recognized as potential delivery platforms for multi‐stage pathological diseases and are promising candidates for next‐generation therapeutic strategies targeting neurodegenerative diseases.

## 2 Results and Discussion

### 2.1 Synthesis and Characterization of poly(OEGMA‐co‐AEMA)‐*g*‐Caffeic Acid (pOAC) and Dialdehyde Poloxamer 407 (DPX)

**Figure 2:**
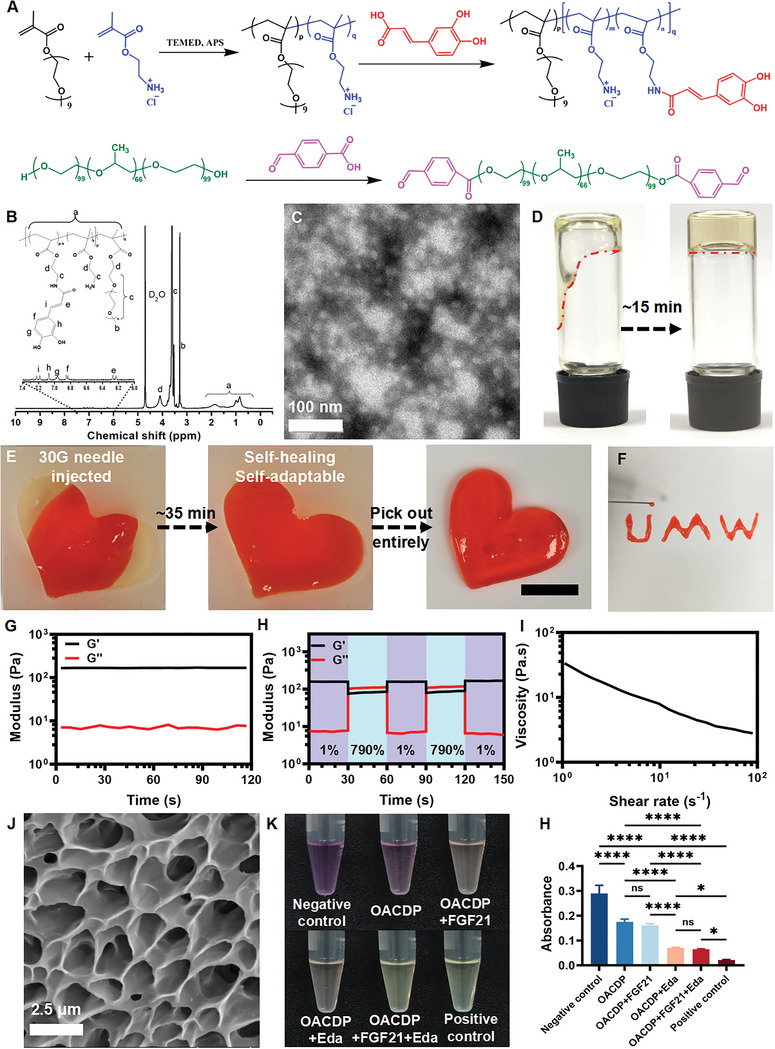
Synthetic chemistry, preparation, morphology, and characterization of the OACDP hydrogel. A) The synthesis routes of pOAC as the main chain and DPX as the nano micellar crosslinker. B) The ^1^H NMR spectrum of pOAC. C) The TEM image of DPX. D) The macroscopic gelation process of OACDP hydrogel. E) The OACDP hydrogels can be injected through 30‐gauge syringe needles with 150 µm internal diameter into the heart‐shaped mold. An integrated heart‐shaped hydrogel with a smooth and homogenous appearance formed after self‐adaption and self‐healing for 30 min at 25 °C. The scale bar represents 1 cm. F) The hydrogels can be injected through 30‐gauge syringe needles to write letters. G) Time‐sweep tests of the OACDP hydrogel after complete gelling show the steady storage moduli (G’) and loss moduli (G’’) of the hydrogels at 1 Hz frequency and 1% dynamic strain. H) Self‐healing properties of the OACDP hydrogels were investigated through the continuous damage‐healing cycles at alternate 1 and 790% dynamic strains, respectively. Each step was processed for 30 s at 1 Hz frequency. I) Shear‐thinning behavior of the OACDP hydrogels was determined by measuring the steady shear viscosity versus shear rate. J) The SEM image for the cross‐section of the OACDP hydrogel. The free radical scavenging capacity of hydrogels was evaluated via DPPH solution. H) Photos for color changes of DPPH solution (Negative control) after free radical scavenging by various groups of hydrogels, including OACDP hydrogel, OACDP hydrogel with FGF21, OACDP hydrogel with Eda, OACDP hydrogel with FGF21 and Eda, and the positive control (ascorbic acid). I) The absorbance of the DPPH solution in each group was detected via UV–vis. Data are represented as mean ± SD (n ≥ 3). ^*^ *p* < 0.05 and ^****^ *p* < 0.0001 between the indicated groups.

Poly(OEGMA‐co‐AEMA) grafted with caffeic acid (CA) was prepared via aqueous free‐radical polymerization and amidation as shown in **Figure** **2**A. Initially, the binary copolymer poly(OEGMA‐co‐AEMA) (molecular weight of ≈30.66 kDa) was prepared via aqueous free‐radical polymerization of poly(ethylene glycol) methyl ether methacrylate (OEGMA) and 2‐aminoethyl methacrylate hydrochloride (monomers) using *N, N, N′, N′*‐tetramethylethylenediamine (TEMED) and ammonium persulfate (APS) as initiators. Subsequently, CA was grafted onto poly(OEGMA‐co‐AEMA) through an amidation reaction between the carboxyl groups of CA and the amino groups on the side chains of the copolymer. This reaction resulted in the formation of a hydrogel with hydrophilic functional groups in the main chain. As shown in the ^1^H nuclear magnetic resonance spectroscopic (NMR) spectra in Figure 2B and Figure S1 (Supporting Information), new characteristic peaks in the low field region (7.4–6.0 ppm) attributed to CA appeared for the poly(OEGMA‐*co*‐AEMA)‐*g*‐caffeic acid (pOAC) polymer, indicating that CA was successfully grafted onto the polymer side chains. Based on the spectra, the amino grafting rate of CA was estimated to be ≈21.4%. The Fourier‐transform infrared spectroscopic (FTIR) spectra shown in Figure S2 (Supporting Information) validated the successful synthesis of pOAC. Furthermore, the dialdehyde Poloxamer 407 (DPX) was prepared by modifying both ends of DPX through an esterification reaction with 4‐formylbenzoic acid (Figure 2A). Amphiphilic DPX could self‐assemble in an aqueous solution to form micelles with a diameter of ≈9.9 nm (Figure 2C). The ^1^H NMR data (Figure S3, Supporting Information) of DPX and the morphological features of the self‐assembled nanospheres were consistent with those reported in our previous study.^[^ ^20a^ ^]^ The zeta potentials of pOAC and DPX were 33.4 ± 0.7 and −6.7 ± 1.2 mV, respectively. The hydrodynamic diameter of DPX was 12.3 ± 2.7 nm, which was slightly larger than that calculated from the transmission electron microscopic (TEM) image. These results were consistent with those of our previous study.^[^ ^20^ ^]^

### 2.2 Preparation, Optimization, and Characterization of pOAC/DPX (OACDP) Hydrogels

A self‐healing bioactive hydrogel (named OACDP) was constructed via a Schiff base reaction between the amino groups of the main chain of pOAC and the aldehyde groups of DPX nanospheres. As shown in Figure 2D, the mixture of pOAC polymers and DPX nanospheres exhibiting a solid state indicated the successful synthesis of the OACDP hydrogel. The optimization of OACDP hydrogel was well prepared. The formulae and basic properties of the hydrogels are summarized in Table S3 (Supporting Information). The maximum concentration of pOAC dissolved in deionized (DI) water or phosphate buffered saline (PBS) was 10 wt%, and the optimal concentration of DPX for producing the hydrogel was 5 wt% (final concentration in the hydrogel).^[^ ^20^ ^]^ Studies have reported that a relatively soft matrix with a stiffness ranging from 0.1 to 1 kPa provides a favorable environment for the neural tissue.^[^ ^21^ ^]^ Only OACDP hydrogels exhibited a sufficient modulus (≈167 Pa) and a gel state. When the concentration of pOAC was < 3 wt% (i.e., OACDP1, OACDP2, and OACDP3), the hydrogels exhibited a fluid‐like state as evidenced by a low modulus (< 50 Pa) and a tendency to flow with rapid self‐healing (within 2 min). Moreover, the swelling ratio of these hydrogels could not be measured because of their disintegration upon swelling in PBS. OACDP4 hydrogels showed an improper modulus (≈72 Pa) and a high swelling ratio (≈150%), indicating their incompatibility for use in rat models of PD. Consequently, the OACDP hydrogel composed of 5 wt% pOAC and 5 wt% DPX were selected for further experiments. After 15 min of crosslinking at room temperature, the mixture of 5 wt% pOAC and 5 wt% DPX transformed into a gel state. The prepared OACDP hydrogel exhibited satisfactory self‐healing and injectable properties. The hydrogel was extruded into a heart‐shaped mold through a 30‐gauge needle, resulting in the formation of a smooth and intact gel within 35 min (Figure 2E). Additionally, as shown in Figure 2F, the OACDP hydrogel could be used to write the letters through extrusion. These macroscopic observations validated the remarkable injectability, self‐adaptability, and self‐healing properties of the OACDP hydrogel.

The mechanical properties of the OACDP hydrogel were evaluated using a rheometer at room temperature. As shown in Figure 2G, the storage modulus (G′) of the hydrogel was ≈170 Pa, which indicated that the gel had appropriate mechanical stability for brain tissue.^[^ ^21^ ^]^ Figure S4 (Supporting Information) shows variations in the modulus of the OACDP hydrogels at a shear strain amplitude of 0.1–800% and a frequency of 1 Hz. The gel‐to‐sol conversion occurred at a strain amplitude of ≈765%. The OACDP hydrogels demonstrated reversible gel‐sol‐gel transitions in successive damage–healing cycles at alternating strains of 1 and 790% (Figure 2H). At the larger shear strain amplitude (790%), the hydrogel converted from a gel‐like state (G′ over G″) to a sol‐like state (G″ over G′). At the lower shear strain amplitude (1%), the hydrogel reverted to the initial gel state (Figure 2I). Furthermore, the viscosity of the OACDP hydrogel steadily decreased with an increase in the shear rate, which suggested that the hydrogel had shear‐thinning properties and favorable injectability. The morphological features of the OACDP hydrogel were examined using a scanning electron microscope (SEM). As shown in Figure 2J, the hydrogel had a homogeneous porous structure with an average pore size of 2.9 ± 0.5 µm. In vitro degradation experiments showed that ≈15% of the hydrogel remained at the application site after 14 days (Figure S5, Supporting Information). Studies have demonstrated that hydrogel degradation is more rapid in vivo than in vitro.^[^ ^22^ ^]^ Therefore, the duration of hydrogel application was set to 14 days for subsequent animal experiments.

ROS scavenging assay (Figure 2K,H) showed that compared with the negative‐control group (only DPPH solution), the OACDP hydrogel, OACDP hydrogel loaded with FGF21, OACDP hydrogel loaded with Eda, and OACDP hydrogel loaded with both FGF21 and Eda exhibited lighter shades of purple, with a decrease in the characteristic absorption of DPPH being observed at 517 nm. These results suggested that all hydrogel groups possessed ROS‐scavenging properties, which may be attributed to the presence of CA.^[^ ^23^ ^]^ In particular, the OACDP hydrogel loaded with both FGF21 and Eda and that loaded with only Eda showed the lightest purple color, indicating the best antioxidant activity. These results indicated that Eda enhanced the antioxidant capability of the OACDP hydrogel.^[^ ^24^ ^]^

### 2.3 Kinetics of Model Drug Release

**Figure 3:**
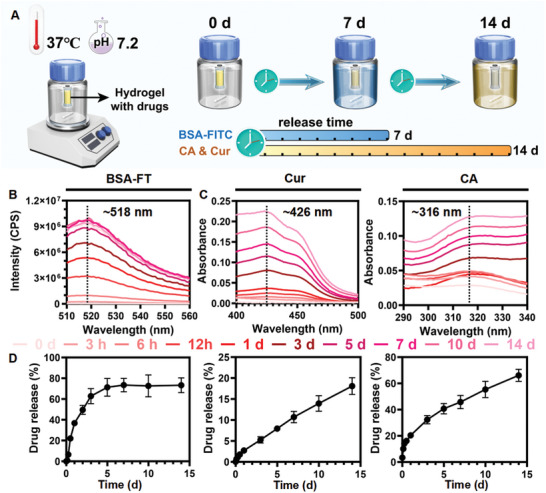
In vitro time‐dependent triple drug release evaluation in PBST buffer at 37 °C. A) The schematic diagram of OACDP hydrogel containing three drugs, including bovine serum albumin‐fluorescein isothiocyanate (BSA‐FT, hydrophilic protein drug tracer), curcumin (Cur, hydrophobic drug tracer), and CA (bioactive molecule), placed in a commercial dialysis tube. The release time of three drugs was demonstrated. B) The fluorescent spectrum of BSA‐FITC, together with (C) UV‐vis spectra of Cur and CA, were obtained from the tracer‐containing buffers at different time points. D) The release profiles of various tracers were calculated from the spectra. Data are represented as mean ± SD (n ≥ 3).

As a localized advanced TDDS, the OACDP hydrogel simultaneously delivered a hydrophilic protein drug, a hydrophobic clinical drug, and a bioactive molecule to the brain owing to its unique hierarchical structure. Because the three drugs were loaded into the hydrogel through different mechanisms, we speculated that the hydrogel might exhibit different drug release kinetics and mechanisms. As shown in **Figure** **3**A, drug release was assessed in vitro using a commercial dialysis device, which allowed the protein drug (60–70 kDa) to pass through the dialysis membrane (MWCO 100 kDa). Fluorescence spectroscopic (Figure 3B) and UV–vis spectroscopic (Figure 3C) data were collected for samples at different time points from the surrounding buffer, and the three datasets were subsequently converted to corresponding drug release curves (Figure 3D). The curves were modeled using three common mathematical models (Equations (4), (5), (6)), namely, zero‐order, first‐order, and Korsmeyer–Peppas (KP). The results of curve‐fitting are shown in **Table** **1**. An average R^2^ value of > 0.95 indicated a good fit, suggesting that the regression model was reliable.

**Table 1:** The fitting mathematical models with the corresponding parameters and R^2^ values.

Tracer	Model	R^2^	K
BSA‐FT (hydrophilic protein model drug)	Zero‐order	0.586	5.208
	First‐order	0.993	0.618
	Korsmeyer‐Peppas	0.880	0.357
Cur (hydrophobic clinic model drug)	Zero‐order	0.990	1.278
	First‐order	0.887	0.680
	Korsmeyer‐Peppas	0.998	0.759
CA (bioactive molecule)	Zero‐order	0.929	4.171
	First‐order	0.890	0.261
	Korsmeyer‐Peppas	0.992	0.522

Hydrophilic drugs encapsulated in nondegradable hydrogels are usually delivered through Fickian diffusion.^[^ ^25^ ^]^ However, in this study, the hydrophilic protein drug encapsulated in the OACDP hydrogel exhibited first‐order release kinetics, and the inability of its release curve to fit the KP model might be attributed to the relatively large size of the drug (> 60 kDa). Although the drug release duration was shorter (peaking at 7 days) than that reported in our previous study,^[^ ^20b^ ^]^ the OACDP hydrogel effectively maintained high drug release (≈73%). The release curve of the hydrophobic drug (curcumin, Cur) encapsulated in the OACDP hydrogel showed a good fit to zero‐order and KP models. In the KP model, the fitted n‐value was 0.87, which was close to the critical value (0.89). This finding suggested non‐Fickian diffusion, that is, a combination of drug diffusion and gel network disintegration.^[^ ^26^ ^]^ The zero‐order release kinetics observed for the hydrophobic drug were consistent with those reported in our previous study;^[^ ^20b^ ^]^ however, the amount of drug released at 14 days was higher, which may be attributed to the shorter structure of the main chain in the OACDP hydrogel. The release curve of the bioactive molecule CA integrated into the main chain of the OACDP hydrogel showed a good fit only to the KP model, showing non‐Fickian diffusion (n = 0.77). A small plateau phenomenon was observed within 24 h, possibly owing to the dynamic equilibration of Schiff base linkages in the hydrogel. A large amount of CA (≈66%) was released after 14 days, which may be attributed to the hydrophilic nature of CA and the degradation capability of the hydrogel. The innovative TDDS developed in this study can be modified to achieve asynchronous, stimulatory, or emergent drug release based on the pathological characteristics of the disease.

### 2.4 In Vitro Cell Experiments

**Figure 4:**
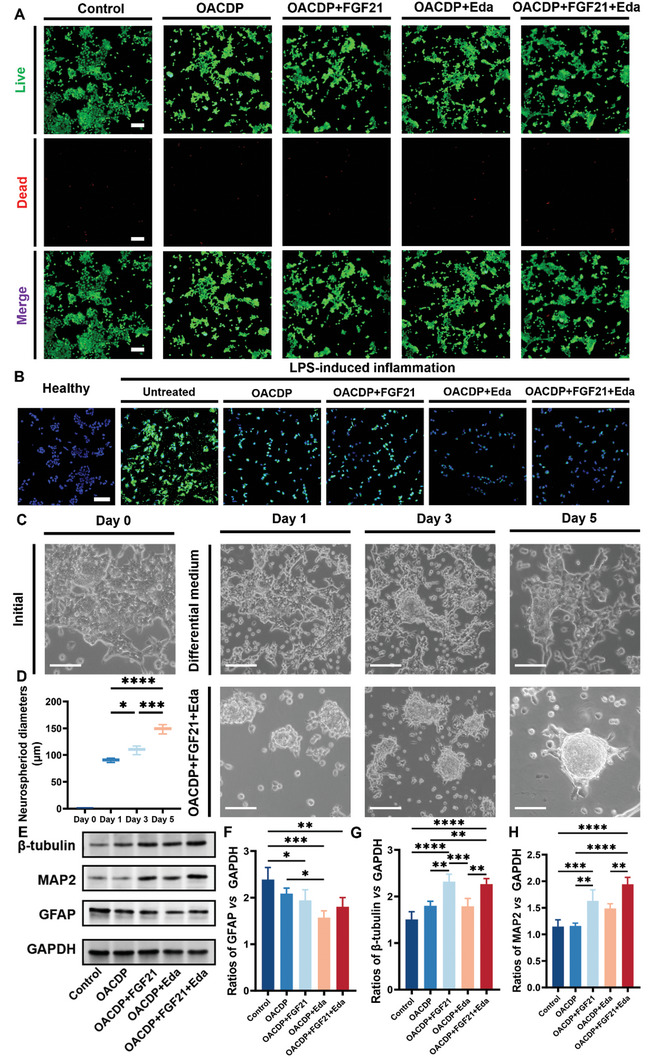
Viability, morphology, and differentiation of NSCs. A) The microscopic fluorescent images of NSCs with live/dead staining in each group after 12 h of cultivation. The scale bar represents 150 µm. Live cells, green; dead cells, red. B) ROS visualization of microscopic fluorescent images in LPS‐induced NSCs and each group treatment by DCFH‐DA (ROS fluorescent probe) staining. The scale bar represents 100 µm. C) The morphology and behavior of NSCs cultured with differential medium and OACDP hydrogel containing FGF21 and Eda aggregated into the neurospheroids on day 0, day 1, day 3, and day 5, followed with the diameter of the neurospheroids being counted (D). The scale bar represents 100 µm. E) Western blot (WB) analysis shows the protein expressions in NSCs after incubation with differential medium (control), OACDP hydrogel, OACDP hydrogel loaded FGF21, OACDP hydrogel loaded Eda, and OACDP hydrogel loaded FGF21 and Eda for 5 days. Densitometric analysis for the protein expression of (F) GFAP, G) β‐tubulin and H) MAP2 normalized to that of GAPDH. Data are represented as mean ± SD (n ≥ 3). *p < 0.05, **p < 0.01, ***p < 0.001, and ****p < 0.0001 between the indicated groups.

All cell experiments were performed using Transwell chambers in 24‐well plates. Cell viability was analyzed via cell counting kit‐8 (CCK‐8) assay after 1 day of culture (Figure S6, Supporting Information). All groups showed high cell viability (> 95%), indicating that the OACDP hydrogel was biocompatible. A live/dead assay was performed to verify cell viability, and the results were consistent with those of the CCK‐8 assay (**Figure** **4**A; Figure S7, Supporting Information). After the cells were treated with lipopolysaccharide (LPS), the production of intracellular ROS was determined using a commercial ROS kit (Figure 4B). The fluorescence intensity was remarkably lower in the hydrogel‐treated groups than in the LPS‐treated group (> 70% reduction; Figure S8, Supporting Information). Although the cells in the OACDP + Eda and OACDP + FGF21 + Eda groups showed the optimal clearance of endogenous ROS, the levels of ROS in neither group (fluorescent intensity ≈3) were restored to those in control NE‐4C cells. The ability of CA to scavenge ROS is mainly driven by its phenolic hydroxyl group.^[^ ^23^ ^]^ Eda, as the first described free radical scavenger,^[^ ^27^ ^]^ can regulate multiple antioxidant‐related cellular pathways, such as ROS‐NF‐κB,^[^ ^28^ ^]^ and the recovery of mitochondrial superoxide dismutase (SOD) levels.^[^ ^29^ ^]^ The antioxidant ability of the triple‐loaded OACDP hydrogel was markedly superior to that of single drug‐loaded hydrogels in vitro, suggesting that the triple‐loaded hydrogel might exhibit good antioxidant activity in vivo.

During the differentiation of NE‐4C cells, their morphological features were visualized under an optical microscope for 5 days (Figure 4C). The cells gradually aggregated into neurospheroid‐like structures and their diameter increased over time (Figure 4D). The diameter of neurospheroids reached ≈150 µm after 5 days of proliferation, aggregation, and differentiation, exhibiting a 2‐fold increase from the initial cell diameter (recorded on the first day of culture). However, the differentiated cells in the culture plate showed only a slight aggregation and did not form spheroids. Studies have shown that NE‐4C cells exhibit significant spheroidal aggregation when differentiating from neurons.^[^ ^30^ ^]^ Therefore, we used western blot (WB) to confirm the direction of NE‐4C cell differentiation after treating the cells with hydrogels. The results are shown in Figure 4E, and the raw data are provided in Figure S9 (Supporting Information). The expression of the astrocyte‐related protein glial fibrillary acidic protein (GFAP, glial marker) was downregulated in all hydrogel‐treated groups; among them, the most marked decrease was noticed in the OACDP + Eda group compared to the control group (Figure 4F). The OACDP + FGF21 + Eda group, although also showing a clear down‐regulation of GFAP, showed an insignificant decrease compared to the OACDP + Eda group. The expression of the neuron‐associated protein β‐tubulin (an early neuronal marker) (Figure 4G) and microtubule‐associated protein 2 (MAP2, a mature neuronal marker) (Figure 4H) was significantly upregulated in the OACDP + FGF21 and OACDP + FGF21 + Eda groups. The OACDP group, which was treated with only the CA‐containing hydrogel matrix, did not show major changes in cell behavior or morphology, possibly owing to the low concentration of CA in the culture medium after release. Although the OACDP hydrogel loaded with Eda did not significantly promote the differentiation of neural stem cells to neurons, it effectively inhibited their differentiation to astrocytes, which may be attributed to the strong ROS‐scavenging ability of Eda. Neurons require a high oxygen concentration for their physiological activities,^[^ ^31^ ^]^ and astrocytes are stimulated by ROS.^[^ ^32^ ^]^ Both processes are affected by the presence of Eda. FGF21 effectively contributed to neuronal differentiation and Eda inhibited the development of astrocytes, both of which are desired therapeutic effects in PD^[^ ^6b^ ^]^ and are consistent with the findings of a previous study.^[^ ^33^ ^]^ Therefore, the triple drug‐loaded OACDP hydrogels can sustain the bioactivity of FGF21 for further asynchronous release, showing promise as a therapeutic strategy for PD.

### 2.5 Behavioral Evaluation of Rat Models of Parkinson's disease (PD)

**Figure 5:**
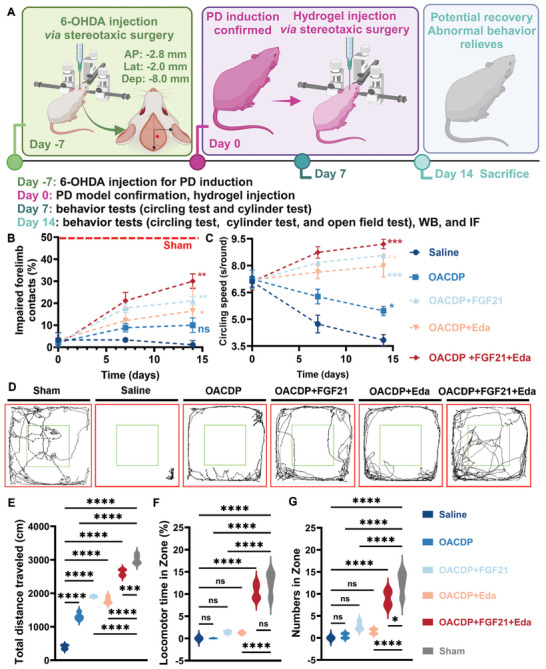
Behavioral evaluation of PD rats treated by hydrogels. A) Schematic illustration for the 6‐OHDA‐induced and hydrogel‐injected PD rat model via stereotaxic surgery. B,C) Quantitative assessment for the functional recovery of different conditions treated PD rats (injected by saline, OACDP hydrogel, OACDP hydrogel loaded FGF21, OACDP hydrogel loaded Eda, or OACDP hydrogel loaded FGF21 and Eda) as compared to the untreated PD rats (0 days), based on the impaired forelimb contact proportion (B) and the left‐side circling speeds (C). *p < 0.05, **p < 0.01, and ***p < 0.001 between the indicated groups versus saline group.(D) Total distance traveled and line crossing in the open field with the center zone. E) Total traveled distance, F) locomotor time in the center zone, and G) entry times in the center zone were counted through Smart 3.0 software. Eight rats per experimental group were used for in vivo experiments. Data are represented as mean ± SD (n ≥ 3). *p < 0.05 and ****p < 0.0001 between the indicated groups.

The therapeutic potential of pristine and drug‐loaded OACDP hydrogels was systematically evaluated in rats with 6‐OHDA‐induced PD (**Figure** **5**A). The rat model of 6‐hydroxydopamine (6‐OHDA)‐induced PD is superior to the classical mouse model of N‐methyl‐4‐phenyl‐1,2,3,6‐tetrahydropyridine‐induced PD in terms of the stability of translational and behavioral symptoms. In addition, 6‐OHDA‐induced PD is more analogous to the progression of neurodegeneration in human PD.^[^ ^15^ ^]^ However, the 6‐OHDA‐induced PD model is rarely used to evaluate the therapeutic efficacy of biomaterial implants because of the complexity of stereotactic techniques and secondary surgeries.^[^ ^34^ ^]^ In this study, the status of motor function recovery in rats with PD treated with drug‐loaded hydrogels was evaluated using three behavioral assays, namely, the cylinder test (Figure 5B), the circling speed test (Figure 5C), and the open field test (Figure 5D). After 14 days of treatment, the contact ratio of the impaired forelimb significantly improved in all hydrogel‐treated groups when compared with the saline‐treated group. Rats in the OACDP + FGF21 group recovered > 20% of forelimb use, whereas those in the OACDP + FGF21 + Eda group recovered >30% of forelimb use, which was significantly higher than that in the OACDP + FGF21 group. Although the forelimb contact ratio of the OACDP + FGF21 + Eda group was lower than that of the sham group (≈50%), behavioral abnormalities were alleviated. This improvement may be closely related to the immediate neuroprotective function of FGF21.^[^ ^18^, ^35^ ^]^ Except for the saline and OACDP hydrogel groups, all other groups showed a significant increase in the time per round of counterclockwise rotation. Rats in the OACDP + FGF21 and OACDP + FGF21 + Eda groups exhibited contralateral rotation behavior to some extent. Rats in the saline and OACDP hydrogel groups exhibited persistent left‐lateral rotation behavior that worsened over time, with the deterioration being significantly slower in the OACDP hydrogel group. As evidenced by the low proportion of use of the impaired forelimb, rats in the saline‐treated group did not show any significant recovery of motor function. The healthy rats in the sham group did not show ipsilateral rotation behavior after apomorphine (APO) injection; therefore, the circling speed of the sham group was not considered for comparison. In the open field test (a general measure of exploratory behavior), total distance traveled (Figure 5E), duration of locomotion in the central area (Figure 5F), and number of entries into the central area (Figure 5G) were evaluated to assess the locomotor activity of rats with PD.^[^ ^36^ ^]^ The time spent in the central area was significantly longer merely in the OACDP + FGF21 + Eda group than the other groups. Compared with rats in the sham group, those in the saline group were more inclined to stay in corners and move around in place, showing anxiety‐like behavior. These results were consistent with those of a previous study.^[^ ^37^ ^]^ Rats in the sham group showed the best performance in all three test parameters. Among rats with PD, those in the OACDP + FGF21 + Eda group explored the central area most actively. Although rats in the OACDP, OACDP + FGF21, and OACDP + Eda groups showed an improvement in exploratory behavior, the difference between these groups and the saline group was not statistically significant. These results indicated that after the immediate neuroprotective effects of FGF21, the sustained inhibition of neuroinflammation by Eda was effective in alleviating behavioral abnormalities in rats with PD.^[^ ^33^, ^38^ ^]^ FGF21 has been shown to alleviate anxiety‐like behavior.^[^ ^39^ ^]^ However, treatment with FGF21‐loaded OACDP hydrogels did not significantly increase the exploratory behavior of rats with PD toward the central area, which may be related to the frequency and amount of the drug administered. On the contrary, treatment with OACDP hydrogels loaded with both FGF21 and Eda significantly improved the exploratory behavior of rats, possibly by exerting neuroprotective effects first and long‐term antioxidant effects later.^[^ ^40^ ^]^ Conductive chitosan hydrogels containing nanogold have been shown to alleviate behavioral abnormalities in PD. This therapeutic effect is attributed to the use of conductive carriers that are more likely to affect neural cells when compared with the use of drugs.^[^ ^41b^ ^]^ In addition, compared with pristine conductive hydrogels, Cur‐loaded conductive hydrogels have been shown to slightly improve behavioral symptoms in rats with PD.^[^ ^15^ ^]^ However, the use of Cur alone is not effective, and the hydrogels do not have an asynchronous release effect, suggesting that a new generation of smart hydrogel carriers is required. Altogether, the results of the three behavioral assays indicated that the efficacy of triple‐loaded OACDP hydrogels with asynchronous drug release was superior to that of single drug‐loaded hydrogels in the treatment of PD.

### 2.6 Neuroprotection in Substantia Nigra Compacta (SNc) and Striatum

**Figure 6:**
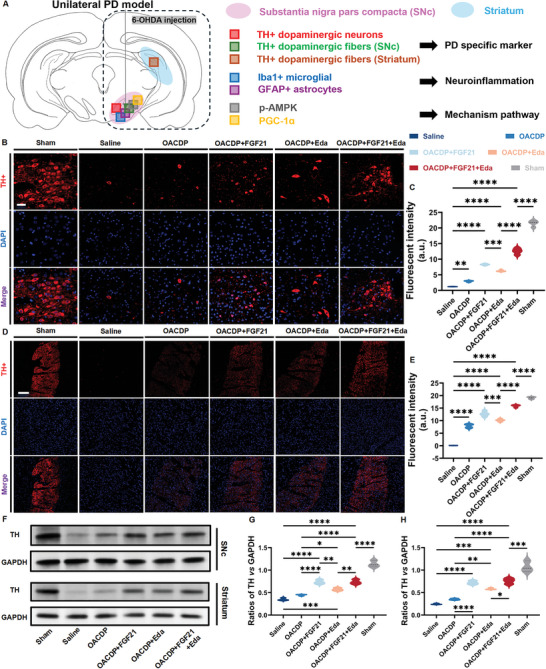
In vivo immunofluorescent and WB analyses for tyrosine hydroxylase (TH, PD specific marker) in SNc and striatum. A) Rat brain atlas showing the locations of the striatum, SNc, and markers of immunostaining for various targets, such as PD functional protection, neuroinflammation, and a potential mechanism pathway. The expression of TH+ dopaminergic neurons in SNc (B) and TH+ dopaminergic fibers in the striatum (D) were investigated and validated 14 days after the second surgery injection. The average fluorescent intensities were quantified and presented as graphics for TH+ dopaminergic neurons in SNc (C) and TH+ dopaminergic fibers in the striatum (E) of each group. The scale bars represent 40 and 200 µm, respectively. F) WB analysis of SNc and striatum shows the TH protein expressions of the sham group (healthy rats), saline group (PD rats), and each hydrogel‐treated group. Densitometric analysis of the TH protein expression for (G) SNc and (H) striatum was normalized to that of GAPDH. Data are represented as mean ± SD (n ≥ 3). *p < 0.05, **p < 0.01, ***p < 0.001, and ****p < 0.0001 between the indicated groups.

The representative regions in and near the substantia nigra compacta (SNc) and the design scheme of the corresponding markers are shown in **Figure** **6**A. Coronal tissues were collected from the site of OACDP injection (4 µL) in rats, and the tissue sections were analyzed via hematoxylin and eosin (H&E) staining (Figure S10, Supporting Information). After 14 days of injection, no hydrogel residue was detected in the coronal tissue sections, suggesting that the injected hydrogels were almost completely degraded. These results were consistent with those of in vitro degradation experiments. Subsequently, histopathological staining was used to evaluate the expression of tyrosine hydroxylase (TH), a characteristic marker associated with PD and one of the rate‐limiting enzymes in dopamine biosynthesis, in the SNc and projective region striatum of rats after 14 days of hydrogel injection. Immunofluorescence images of TH‐positive (TH+) neurons and fibers in the SNc are shown in Figure 6B and Figure S11A (Supporting Information), respectively, and the semi‐quantitative data are summarised in Figure 6C and Figure S11B (Supporting Information), respectively. The number of TH+ neurons in the SNc was significantly higher in the hydrogel‐treated groups than in the saline‐treated group. In particular, the average fluorescence intensity of the OACDP + FGF21 and OACDP + FGF21 + Eda groups (≈8.3 and ≈12.5, respectively) exceeded that of the saline‐treated group (≈1.2) by 6‐fold and 10‐fold, respectively. Similar changes were observed in the number of TH+ dopaminergic neural fibers, with the average fluorescence intensity of the two FGF21‐loaded hydrogel groups (≈3.8 and ≈5.0, respectively) exceeding that of the saline‐treated group (≈1.4) by more than 2.6‐fold. The minor therapeutic effects observed in the OACDP hydrogel group (≈2.9 for TH+ neurons and ≈2.1 for TH+ fibers in SNc) may be attributed to the presence of CA, which has been shown to participate in the regulation of PD.^[^ ^42^ ^]^ A chitosan‐based hydrogel containing polyphenols (tannic acid) has been proven effective in treating PD.^[^ ^15^ ^]^ The limited therapeutic efficacy of the OACDP hydrogel can be primarily attributed to the low amount of CA in the hydrogel matrix and its slow release from the hydrogel, both of which reduce the applicability of the hydrogel as a therapeutic adjunct. A similar trend was observed in the fluorescence intensity of TH+ dopaminergic fibers in the striatum (Figure 6D,E). Overall, the results of immunofluorescence staining for TH were consistent with those of behavioral analyses. Furthermore, WB was performed to visualize and evaluate the protein expression of TH in the SNc and striatal regions of rats (Figure 6F–H). The uncropped images are presented in Figure S13 (Supporting Information). According to statistical analysis, the results of WB were mostly consistent with those of IF. Although the protein expression of TH was considerably high in the OACDP + FGF21 and OACDP + FGF21 + Eda groups (≈0.7 and ≈0.7, respectively), the expression level differed by ≈35% between these groups and the sham group (≈1.1). However, the difference between saline‐treated and OACDP‐treated groups was not statistically significant. According to the results of histopathological analysis, the immediate neuroprotective effects of FGF21 on dopaminergic neurons and fibers are crucial, whereas the subsequent scavenging of ROS by Eda to alleviate neuroinflammation can be considered a composite therapeutic effect. These results validate that triple drug‐loaded OACDP hydrogels with asynchronous drug release can achieve relatively optimal therapeutic efficacy in PD.

### 2.7 Regulation of Neuroinflammation and Mitochondrial Function

**Figure 7:**
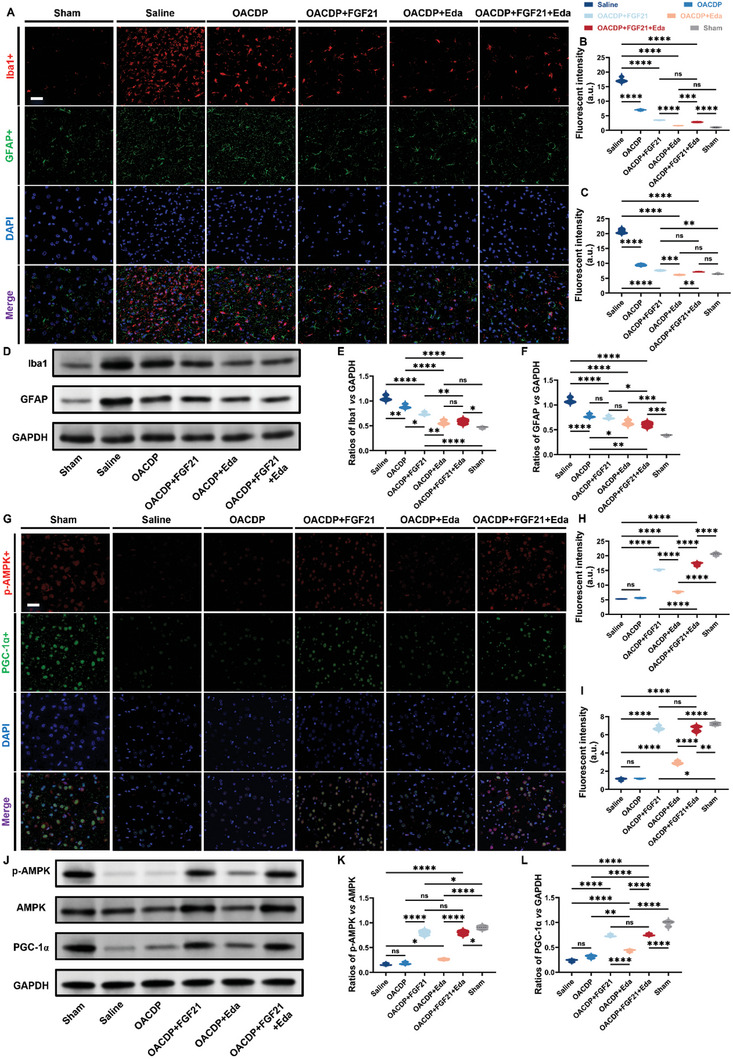
Immunofluorescent staining and WB investigations of neuroinflammatory and mitochondrial dysfunction in SNc. A) Fluorescent images of Iba1+ microglia (red) and GFAP+ astrocytes (green) after implantation in the brain for 14 days. Quantification for the fluorescent intensity of (B) Iba1+ microglia and (C) GFAP+ astrocytes in vivo. D) The visualization of WB analysis of each group and related densitometric analysis of the (E) Iba1 and (F) GFAP expressions normalized to that of GAPDH. G) Fluorescent images of p‐AMPK (red) and PGC‐1α (green) after implantation in the brain for 14 days. Quantification for the fluorescent intensity of (H) p‐AMPK and (I) PGC‐1α in vivo. J) The visualization of WB analysis of each group and related densitometric analysis of the K) p‐AMPK/AMPK and L) PGC‐1α/GAPDH. Both scale bars represent 40 µm. Data are represented as mean ± SD (n ≥ 3). *p < 0.05, **p < 0.01, ***p < 0.001, and ****p < 0.0001 between the indicated groups.

Based on the results of previous studies and the pathological mechanisms of PD,^[^ ^19^, ^35^, ^43^ ^]^ the effects of FGF21‐ and Eda‐encapsulated OACDP hydrogels on neuroinflammation and mitochondrial function were examined in rats with PD. 6‐OHDA induces PD primarily by affecting mitochondrial function and facilitating sustained ROS production to induce neurotoxicity and mitochondrial damage.^[^ ^44^ ^]^ The total SOD activity within the concerned brain region was evaluated using a corresponding SOD assay kit (Figure S13, Supporting Information). The results were found to be consistent with those of the in vitro ROS assay. CA present in OACDP hydrogels exerts potent antioxidant effects, whereas FGF21 does not possess significant antioxidant activity. Among rats with PD, those in the OACDP + FGF21 + Eda group exhibited the optimal antioxidant activity (≈ 46.64 U mg^−1^); however, it was ≈16% lower than that of the sham group (healthy rats) (≈55.76 U mg^−1^). Overall, the combined use of drugs resulted in stronger antioxidant activity when compared with their separate use. The two major cell types associated with neuroinflammation are astrocytes and microglia, both of which are activated during neurotoxicity and neurodegeneration.^[^ ^45^ ^]^ The number of microglia in the brain tissues was significantly lower in all hydrogel‐treated groups (an average fluorescent intensity of < 7.0 for all) than in the saline‐treated group (an average fluorescent intensity of ≈17.3). These results indicated that CA present in the OACDP hydrogel contributed to antioxidant defenses in rats with PD (**Figure** **7**A,B).^[^ ^46^ ^]^ Changes in the number of GFAP+ astrocytes were consistent with those in the number of Iba+ microglia (Figure 7C). Under physiological conditions, astrocytes release neuroprotective growth factors. However, the excessive accumulation of Iba+ microglia in PD produces an acidic inflammatory environment that promotes the development of GFAP+ astrocytes and sustains neuroinflammation, thereby exacerbating the symptoms of PD.^[^ ^47^ ^]^ Among all groups of rats with PD, the OACDP + Eda group showed the best inhibition of neuroinflammation (≈1.6 for Iba1+ microglia and ≈6.2 for GFAP+ astrocytes), which may be attributed to the ability of the Eda‐loaded hydrogel to scavenge ROS and improve circulation.^[^ ^48^ ^]^ Although the combined use of Eda and FGF21 caused a slight decrease in the anti‐neuroinflammatory effect (≈2.8 for Iba1+microglia and ≈7.2 for GFAP+ astrocytes), it exerted favorable anti‐inflammatory effects and superior neuroprotective effects. The protein expression levels of Iba1 and GFAP in rat SNc tissues were visualized and quantified via WB (Figure 7D–F). The uncropped images are shown in Figure S14 (Supporting Information). According to statistical analysis, changes in both proteins were consistent with those observed in IF analysis. Altogether, the triple drug‐loaded OACDP hydrogel effectively scavenged ROS and enhanced SOD activity while alleviating overall neuroinflammation.

Given that FGF21 in the triple‐loaded OACDP hydrogel exerted major neuroprotective effects, we examined the pathways and mechanisms underlying its effects. A study on rat models of PD showed that FGF21 enhanced mitochondrial function by stimulating the AMPK/PGC‐1α axis in the brain, thereby exerting therapeutic effects against PD.^[^ ^35^ ^]^ As shown in Figure 7G, we evaluated changes in the AMPK/PGC‐1α axis in rats with PD via IF staining. The results showed that phosphorylated AMPK (p‐AMPK) and PGC‐1α were significantly downregulated in the brain tissues of saline‐treated rats with PD. However, treatment with FGF21‐loaded hydrogels substantially increased the expression of both proteins in the brain tissues of rats with PD (Figure 7H,I). The OACDP + Eda group also showed a slight increase in the expression of the two proteins, which may be attributed to the ability of Eda‐loaded hydrogels to regulate intracerebral circulation. These results are consistent with those of a previous study.^[^ ^49^ ^]^ Furthermore, the protein expression of p‐AMPK and PGC‐1α in rat brain tissues was validated via WB (Figure 7J). The raw images are shown in Figure S15 (Supporting Information). The protein expression levels of p‐AMPK and PGC‐1α were normalized to those of AMPK and GAPDH, respectively (Figure 7K,J). Although both indexes are still ≈12% and ≈23% off compared to healthy rats (sham group), respectively, a highly remarkable difference compared to PD rats (saline group) has substantiated that the AMPK/PGC‐1α axis is indeed activated. AMPK, an anti‐inflammatory factor, inhibits the NOX‐mediated production of ROS and the NF‐κB‐mediated production of pro‐inflammatory cytokines (e.g., interleukin‐1 and tumor necrosis factor α).^[^ ^50^ ^]^ Upon treatment with FGF21‐loaded hydrogels, FGF21 may induce AMPK activation, thereby reducing the expression of inflammatory cytokines. Altogether, FGF21 may significantly improve mitochondrial function in the neurons of rats with 6‐OHDA‐induced PD and the combined use of FGF21 and Eda may synergistically activate the AMPK/PGC‐1α axis in rats with PD.

The findings of this study collectively suggest that the novel self‐healing OACDP hydrogel encapsulated with FGF21 and Eda exerts therapeutic effects against PD through various mechanisms. First, in vitro experiments validated that treatment with OACDP hydrogels encapsulated with both FGF21 and Eda promoted the differentiation of NE‐4C NSCs to neurons. Second, in vivo experiments showed that treatment with OACDP hydrogels encapsulated with both FGF21 and Eda improved motor function, alleviated anxiety‐like behavior, and prevented the loss of TH in the SNc and striatum of rats with PD. Mechanistically, the combined use of FGF21 and Eda attenuated the 6‐OHDA‐induced activation of astrocytes and microglia, alleviating neuroinflammation and mitigating mitochondrial damage through potent antioxidative effects. In addition, it synergistically stimulated the AMPK/PGC‐1α axis to improve mitochondrial function. These effects may be attributed to the combined use of FGF21, Eda, and CA (grafted in the pristine hydrogel), and their asynchronous release from the OACDP hydrogel. Considerable therapeutic effects were achieved within 14 days of treatment with only 4 µL of FGF21‐ and Eda‐loaded hydrogels. These findings suggest that the triple‐loaded OACDP hydrogel with asynchronous drug release simultaneously targets multiple pathological mechanisms of PD in a prolonged manner, exerting potent therapeutic effects against PD. Therefore, the hydrogel serves as a promising candidate for developing a new generation of therapeutic strategies targeting neurodegenerative diseases.

## 3 Conclusion

In this study, we developed a novel hierarchical self‐healing hydrogel (OACDP) and encapsulated it with FGF21 and Eda to treat PD. This hydrogel asynchronously released three incorporated drugs. The hydrophilic protein drug FGF21 and the bioactive molecule CA (grafted onto the copolymer) were rapidly released in a first‐order manner and best fitted the KP release model, respectively, whereas the hydrophobic drug Eda was released in a sustained zero‐order manner. The OACDP hydrogel had an optimal modulus (∼167 Pa) and exhibited antioxidant properties (>50%), injectability (30‐gauge needle with 150‐µm inner diameter), and shape‐adaptation properties, rendering it appropriate for use in minimally invasive procedures in precision medicine. Upon release from the hydrogel, FGF21 provides immediate neuroprotection, and Eda subsequently alleviates neuroinflammation by long‐term scavenging of ROS. Treatment with OACDP hydrogels loaded with both FGF21 and Eda promoted the differentiation of neural stem cells to neurons in vitro and promoted the recovery of motor function in rats with PD in vivo at a minimal dose of 4 µL. Histological analysis revealed that the FGF21‐ and Eda‐loaded OACDP hydrogel effectively exerted neuroprotective effects by preventing the loss of TH in the SNc and striatal regions (≈10‐fold), scavenging ROS to alleviate mitochondrial damage, inhibiting the activation of astrocytes and microglia, and inducing the activation of the AMPK/PGC‐1α axis to regulate mitochondrial function, which are multiple mechanisms underlying the treatment of PD. The hydrogel‐based smart drug release system developed in this study can realize asynchronous drug release, serving as an innovative treatment strategy for PD and other neurodegenerative diseases.

## 4 Experimental Section

### Synthesis and Characterization of pOAC and DPX

The poly(OEGMA‐co‐AEMA) copolymer was synthesized by aqueous free radical polymerization. Firstly, 2‐aminoethyl methacrylate hydrochloride (AEMA, Mw = 165.62, 360 mg, Aladdin, China) and OEGMA (Mw = 475, 4 g, Aladdin, China) were dissolved in 150 mL ultrapure water. Under nitrogen protection, APS (90 mg ml^−1^, 167 µL, Aladdin, China) and TEMED (100 mg ml^−1^, 304 µL, Aladdin, China) was slowly added into the above solution. Then, the reaction mixture was stirred at room temperature for 18 h. After that, the reaction solution was dialyzed against H_2_O for 2 days to remove unreacted monomers (MWCO = 3500 Da). Finally, the poly(OEGMA‐co‐AEMA) copolymer was obtained by lyophilizing. The structure of poly(OEGMA‐co‐AEMA) copolymer was detected by ^1^H NMR spectrum (BRUKER AV400, 400 MHz), employing D_2_O (Macklin, China) as a solvent and tetramethyl silane as an internal reference, and Fourier Transform Infrared Spectrometer (Nicolet is50, Thermo Fisher, USA).

The pOAC polymer was synthesized by amidation reaction. First, 1.2 g poly(OEGMA‐co‐AEMA) copolymer, 23 mg *N*‐(3‐dimethylaminopropyl)‐*N*‐ethylcarbodiimide hydrochloride crystalline (EDC, Aladdin, China) and 13.8 mg *N*‐hydroxysuccinimide (NHS, Aladdin, China) were dissolved in 5 mL *N, N*‐dimethylformamide (DMF, Aladdin, China) at room temperature. Then, 21.6 mg CA (Sigma–Aldrich, USA) was added to the above mixture. The pH of the reaction mixture was adjusted to 5 with hydrochloric acid. After the reaction mixture was persistently stirred at room temperature for 24 h, the solution was dialyzed against H_2_O with a dialysis membrane (MWCO = 3500 Da) many times. The product was obtained by freeze‐drying the dialysate. The structure of the pOAC polymer was detected by ^1^H NMR spectrum and FTIR.

The DPX crosslinker was synthesized by Steglich esterification. To prepare the DPX nano‐crosslinker, 4‐formylbenzoic acid (0.24 g, 1.6 mmol, Sigma–Aldrich, USA), 4‐(dimethylamino) pyridine (0.025 g, 0.2 mmol, Sigma–Aldrich, USA), *N,N′*‐diisopropylcarbodiimide (0.4 g, 3.2 mmol, Sigma–Aldrich, USA), and Poloxamer 407 (2 g, 0.16 mmol, Sigma–Aldrich, USA) were sequentially added to anhydrous tetrahydrofuran (100 mL). The reaction was stirred at 25 °C for 48 h. The reacted solution was added to diethyl ether (300 mL). The white precipitate was filtered and dissolved into tetrahydrofuran (100 mL). The solid product was purified by repeating the above‐mentioned precipitation/dissolution cycles. The chemical structure of DPX was detected by ^1^H NMR spectrum. In addition, the amphiphilic DPX polymer could self‐assemble in an aqueous solution to form micelles, as reported in our previous works.^[^ ^20^ ^]^ The zeta potential and hydrodynamic diameter were calculated by a Nano Particle Size and Zeta Potential Analyzer (Zetasizer ultra, Malvern, UK).

### Preparation, Physicochemical Properties, and Rheological Analysis of OACDP Hydrogels

Based on the Schiff base reactions between amino and aldehyde groups, the composite OACDP hydrogel was prepared by mixing pOAC polymer and DPX micelles. The pOAC polymer was dissolved in PBS buffer to prepare main chain solutions at 2, 4, 6, 8, and 10 wt%. The powder of the crosslinker (DPX) was dissolved in PBS buffer to prepare the crosslinker solutions (10 wt%). Then 500 µL DPX solution was added into pOAC solution under vortex at 25 °C to obtain a homogeneous precursor, resulting in the formation of OACDP hydrogel with simultaneous crosslinking.

The porosity was examined by the ethanol soaking method. The porosity value was implemented by the Equation (1).

(1) $$\mathrm{porosity}=\frac{W_{1}-W_{0}}{\rho V}\;\times 100\%$$

where *W_1_* is the wet weight of the rehydrated hydrogel in ethanol, *W_0_* is the solid weight of the hydrogel, *ρ* is the density of ethanol, and *V* is the volume of the hydrogel.

The swelling ratio of the hydrogels was calculated by weighing the wet hydrogel sample after swelling for 24 h, using the following Equation (2):

(2) $$\mathrm{swelling}\;\mathrm{ratio}=\frac{W_{s}}{W_{i}}\;\times 100\%$$

where Ws and Wi represented the wet weight of the well‐swelling hydrogel sample and the initial hydrogel, respectively.

The porous network structure was observed by SEM (operating at 3 kV, TM3000, Hitachi) in the inner cross‐section of lyophilized hydrogels. The in vitro degradation of hydrogels was estimated by tracking the residual mass over time. The soaked hydrogels were retrieved from the PBS buffer at defined time points, rinsed with DI water, and then freeze‐dried. The remaining weight percentage was calculated by the Equation (3):

(3) $$\mathrm{remaining}\;\mathrm{weight}=\frac{W^{\prime \prime }}{W^{\prime }}\;\times 100\%$$

where *W’’* is the initial dried weight of the hydrogel and *W’* is the dried weight of the hydrogel after incubation in pH = 7.4 PBS at 37 °C at defined time points.

The rheological properties of the OACDP hydrogels were estimated at 25 °C using a rheometer (HR‐2, Discovery Series Hybrid Rheometer, TA Instruments) with a cone and plate geometry of 40 mm diameter and a cone angle of 2°. The measurements of storage modulus (G′) and loss modulus (G″) were carried out at a frequency of 1 Hz and a dynamic strain of 1%. The dynamic strain sweep experiments of the hydrogels were investigated at a frequency of 1 Hz in the range of 0.1–1000% dynamic strain amplitudes. The self‐healing properties of equilibrium hydrogels were assessed by rheological experiments with continuous and alternating high strain (790%) and low strain (1%) as damage‐healing cycles at a frequency of 1 Hz. The steady shear test was utilized to characterize the shear thinning behavior of the hydrogels, identified by measuring the viscosity in relation to the shear rate.

### Drug Encapsulation and In Vitro Triple‐Drug Release Experiments of Hierarchical Bioactive OACDP Hydrogels

For the preparation of dual drug‐loaded OACDP hydrogels, hydrophobic drugs need to be loaded in DPX micellar micelles by membrane hydration method.^[^ ^51^ ^]^ The concentrations used for drug encapsulation within the hydrogel were followed from the literature of previous studies.^[^ ^20^, ^52^ ^]^ 2 mg of Cur (model drug, 99.8%, Sigma–Aldrich, USA) or Eda (assay 99%, Sigma–Aldrich, USA) was dissolved in 50 mL of anhydrous ethanol along with 100 mg of DPX powder. The ethanol solution was withdrawn from the system by a rotary evaporator (RE100‐S, DLAB, China) at 12 mbar pressure and dried for 4 h. The obtained DPX/drug powder was redissolved into PBS buffer by sonication to prepare drug‐encapsulated micellar solutions. FGF21 (China Gene Medicine Valley, China) or BSA‐FT (Solarbio, China) were dissolved in the pOAC solution at a concentration of 200 ng mL^−1^ to obtain a final PBS solution of 10 wt% pOAC. Dual‐drug‐loaded OACDP hydrogels were prepared by mixing the pOAC solution containing the protein drug and the DPX solution encapsulated with the hydrophobic drug with reference to the preparation of OACDP hydrogels in the previous section “Preparation, Physicochemical Properties, and Rheological Analysis of OACDP Hydrogels” of Experimental Section.

The hydrophobic clinic drug Cur and hydrophilic protein drug BSA‐FT were selected as the model drugs because the obvious UV–vis absorption and fluorescent intensity, respectively, were beneficial to trace the concentration in the PBST buffer (2% Tween 20 in PBS, pH≈7.2). The DPX and pOAC were firstly mixed with Cur (0.2 wt% in 10 wt% DPX) and BSA‐FT (200 ng mL^−1^ in 10 wt% pOAC), respectively. These two pre‐solutions were then mixed and filled into a commercial dialysis device (Float‐A‐Lyzer, MWCO 100 kDa, Spectrum Laboratories, Inc., USA). The prepared drug‐loaded OACDP hydrogel in the delivery device was dialyzed at 37 °C versus PBST buffer in a glass bottle with a volume of 100 mL. At the predetermined time (3, 6, 12 h, 1, 3, 5, 7, 10, and 14 d), 1.0 mL of the drug‐containing buffer was removed, and another 1.0 mL fresh buffer was supplemented to maintain the total volume of buffer. The amounts of released model drugs were quantified by using the UV–vis spectrometer (Evolution 350, Thermo Fisher, USA) and the fluorescence spectrophotometer (FluoroMax‐4, Horiba Scientific, Japan). Three target drugs or bioactive substances were detected in the buffer including BSA‐FT, Cur, and CA. The characteristic peaks of BSA‐FT, Cur, and CA were observed at ≈518, ≈426, and ≈316 nm, individually.

The following three mathematic models were utilized to identify the drug release kinetics. The first model was the zero‐order model represented by the following Equation (4):

(4) $$Q_{0}-\;Q_{t}=K_{0}\;t$$

where the *Q_0_* and *Q_t_* are the amount of drug in the buffer in the initial time and time t. *K_0_* is the zero‐order release rate constant. And the first‐order model was represented by Equation (5):

(5) $$C_{t}=C_{0}\;e^{-K_{1}t}$$

where the *C_0_* and *C_t_* are the concentrations of the drug in the buffer in the initial time and time t. *K_1_* is the first‐order release rate constant. In addition, the Korsmeyer–Peppas model was represented by Equation (6):

(6) $$\frac{M_{t}}{M_{\infty }}=K_{kp}\;t^{n}$$

where the *M_0_* and *M_t_* are the mass of the drug released in the buffer over time t and the time approaching infinity (the upper limit is the total mass of the drug carried in the hydrogel system). *K_kp_* is a constant incorporated with structural/geometrical information of the delivery system and drug, and n is the release exponent.

### Viability, Scavenging ROS, Morphology, and Differentiation of Neural Stem Cells (NSCs)

The NE‐4C cells, a NSC line that was isolated from the cerebral vesicles, were purchased from Wuhan Sunncell Biotechnology Co. Ltd. NE‐4C were cultured and passaged in Eagle's Minimum Essential Medium (EMEM, Gibco, USA) containing 10% fetal bovine serum (FBS, Sigma–Aldrich, USA) and 1% penicillin–streptomycin antibiotic (PS, Gibco, USA). All in vitro cell experiments were operated with Transwells (Costar, USA). NE‐4C cells were seeded and attached to the 24‐well plate, followed by placing hydrogel‐containing or drug‐loaded hydrogel‐containing Transwells in the 24‐well plate for 24 h. The CCK‐8 (Sigma–Aldrich, USA) assay was utilized to evaluate the cell viability and the cytotoxicity of all hydrogels. The measurement with the CCK‐8 assay was performed at a wavelength of 450 nm using a microplate reader (Mutiskan SkyHigh 1550, Thermo Fisher, USA). The cell viability of all hydrogel groups was normalized to the control group, i.e., cells with culture medium only. The live/dead cell imaging analysis was performed with the Live/Dead Cell Vitality Kits (C2015 M, Beyotime, China). The experiment method was similar to the cell viability test, except for 12‐h co‐incubation. Visualization of dead/live staining tests was performed by confocal laser scanning microscopy (Axio Observer 7, Zeiss, Germany) excited at 488 and 514 nm. The quantification was analyzed using ImageJ software.

The capability of scavenging ROS was determined using a ROS Assay Kit (Beyotime, China). After induction of inflammation using lipopolysaccharide (1 mg mL^−1^, L2630, Sigma–Aldrich, Germany) with NE‐4C cells for 24 h, each group of hydrogels was co‐incubated with inflamed NE‐4C for 24 h. The treated NE‐4C cells were fluorescently stained using 10 µm DCFH‐DA (ROS fluorescent probe) at 37 °C for 20 min and imaged via a laser scanning confocal microscope (LSM 980 with Airyscan2, Zeiss, Germany). The positive and negative control groups were healthy and inflamed NE‐4C cells, respectively. The quantification was analyzed using ImageJ software.

The cell differentiation medium was changed at 70% full growth of NE‐4C cells to test the ability of hydrogel to promote stem cell differentiation. Cell differentiation medium was mainly composed of Neurobasal‐A (Gibco, USA) supplemented with 0.24 mg mL^−1^ GlutaMAX (Gibco, USA), 2% B‐27 Without VitA (Gibco, USA), and 1% PS (Gibco, USA). The experiment method was similar to the cell viability test, except for using a differentiation medium and co‐incubation of 5 days. Cell morphology during differentiation was continuously recorded by light microscopy paired with a charge coupled device camera (AE2000, Yongxin, China). WB method was employed to investigate the protein expression of differentiated stem cells. Differentiated cells were vortexed and shaken after the addition of lysate, placed on ice and repeatedly blown, and vortexed every 10 min. The extracts were then centrifuged at 12000 rpm for 10 min, and the supernatant was collected for protein determination. Protein concentration was estimated using a Bicinchoninic Acid (BCA) Protein Assay Kit (WB6501, NCM, China). Electrophoresis was performed using 40 µg of protein per lane on a 15% gel. Proteins were electrophoresed and transferred to a PVDF membrane (IPVH00010, Immobilon‐P, Millipore, USA). The membrane was then closed with 5% skimmed milk for 2 h and incubated with primary antibodies, including GFAP (ab7260, Abcam, UK, 1:10000, ≈55 kDa), MAP2 (13‐1500, Thermo Fisher, USA, 1:500, ≈58 kDa), Β‐III‐tubulin (ab18207, Abcam, UK, 1 µg mL^−1^, ≈52 kDa), and GAPDH (ab263962, Abcam, UK, 1:1000, ≈37 kDa) at 4 °C overnight. After multiple washes with TBST, the membrane was incubated with horseradish peroxidase (HRP)‐labeled secondary antibody (HRP‐labeled Goat Anti‐Rabbit IgG(H+L) (A0208, Beyotime, China, 1:1000), HRP‐labeled Goat Anti‐Mouse IgG(H+L) (A0216, Beyotime, China, 1:1000)) for 2 h at room temperature. Signals were measured using a ChemiDoc XRS + Imaging System (Bio‐Rad, USA), and then band densities were quantified using ImageJ software.

### In Vivo Rat Model of PD and the Stereotaxic Injection of Hydrogels

Adult male Sprague Dawley (SD) rats (Beijing Vital River Laboratory Animal Technology Co. Ltd., China) weighing 250–350 g was used for animal experiments in this study. All animal experimental procedures in this study were authorized by the Institutional Animal Care and Use Committee (IACUC) of the Oujiang Laboratory (Approval No. OJLAB23112504) and were in accordance with the Animal Care and Use Guidelines Laboratory Animals. When housed in a constant temperature and humidity environment with a 12‐h light/dark cycle, rats were allowed to eat and drink freely. The protocol for inducing PD in rats by microinjection of 6‐OHDA (a neurotoxin, Sigma–Aldrich, USA) into the unilateral medial forebrain bundle (mfb) had been previously reported in the literature.^[^ ^53^ ^]^

Rats were anesthetized with a veterinary anesthetic (Zoletil 50, 40 mg kg^−1^, Virbac, France) and mounted in a stereotactic frame (RWD, China). 6‐OHDA (≈0.023 mg kg^−1^, 4 µL each, 2 µg µL^−1^ in 0.2 wt% ascorbic acid saline solution) was injected using 30‐gauge Hamilton microinjectors. Based on Paxinos and Watson's Rat Brain Mapping Coordination,^[^ ^54^ ^]^ the injection site was chosen at an anterior‐posterior (AP) −2.8 mm × lateral (LAT) −2.0 mm × depth (DEP) −8.0 mm for a rate of 0.5 µL min^−1^. A further 5 min was necessary before withdrawal of the injection cannula to minimize loss of fluid trailing along the injection tract. The rotational behavior of all rats was assayed by additional injections of APO (0.05 mg kg^−1^ s.c., 5 mg mL^−1^ dissolved in 0.2 wt% ascorbic acid saline solution, Sigma–Aldrich, USA) to determine the efficiency of the lesions in the model.^[^ ^41^ ^]^ A metric of successful PD induction was that after mfb injection of 6‐OHDA, PD rats swiveled more than 25 times every 5 min, always turning to the contralateral side of the lesion.

Rats with successfully induced PD identified by behavioral tests were elected to be injected unilaterally with saline and hydrogels. Anesthetized rats were treated with Zoletil 50 and fixed their heads to the stereotaxic apparatus (RWD, China). The injection position was consistent with the lesion site. 4 µL of saline, OACDP hydrogel, OACDP+FGF21 hydrogel, OACDP+Eda hydrogel or OACDP+FGF21+Eda hydrogel was injected at a rate of 0.5 µL min^−1^ through a Hamilton microsyringe with a 26‐gauge needle. After a delay of 5 min, the needle was withdrawn slowly to prevent reflux. Eight rats per experimental group were used for the investigation including the behavioral test, histology analysis, and protein expression evaluation. In total, 48 rats were used for the experiments. A sham‐control group, i.e., healthy rats injected with saline instead of 6‐OHDA in the first operation stage, was employed for better comparison.

### Behavioral Tests of PD Rats

The behavioral experiments were operated according to previous studies with slight modifications.^[^ ^55^ ^]^ The circling behavior of rats was tested by subcutaneous injection of APO on days 7 and 14. The time required for 30 turns of spontaneous rotational behavior in rats was recorded and the average speed per turn of rotation was further calculated to assess the reduction of PD symptoms. The cylinder asymmetry test was also used to study forelimb lateralization in PD rats except for no APO usage. Rats were placed in a glass cylinder with a diameter of 22 cm and a height of 26 cm, and videotaped for 5 min after the rats first contacted the wall of the cylinder with either the damaged or undamaged forelimb, and the number of contacts between the two forelimbs was counted to facilitate comparison with healthy rats. The open‐field experiments were conducted based on previous studies with slight modifications to assess the autonomous and exploratory behaviors of PD rats in a new environment.^[^ ^56^ ^]^ Rats were individually placed in the corner of a gray plastic box (200 × 200 × 50 cm) that served as the open field. A video camera was used to record the locomotion behavior of the rats within the field for 5 min, which was later analyzed using an open‐field experimental video analysis system (Smart 3.0, Panlab SMART video tracking system, Barcelona, Spain). The center area was defined as a square area (100 × 100 cm) situated 50 cm from both edges. The floor of the arena was cleaned between trials with a 10% ethanol solution.

### Hematoxylin and Eosin (H&E) and Immunofluorescent Staining

PD rats were sacrificed after 14 days, and the hearts were then perfused with saline and then fixed in 4 wt% paraformaldehyde (PFA) for further staining analysis. Brains were harvested and immersed in 4 wt% PFA overnight for secondary fixation. The brains were dehydrated, paraffin‐wrapped, and sectioned into 4 µm slices. After deparaffinization, the sections were rinsed with PBST and then blocked with 2 wt% BSA solution for 30 min. As for H&E staining (standard type; Jiangsu KeyGen Biotech Co., Ltd., Nanjing, Jiangsu Province, China), the staining was operated according to the manufacturer's instructions only for the group of OACDP hydrogel‐treated PD rats. The H&E‐stained sections were visualized under optical microscopy (Olympus, Tokyo, Japan).

For immunofluorescent staining, tissue sections were incubated overnight at 4 °C with various primary antibodies for different purposes, including in vivo biocompatibility and histological assessment of PD. Primary antibodies used in this study included anti‐tyrosine hydroxylase rabbit pAb (1:300, GB11181, Servicebio, China), anti‐Iba1 mouse mAb (1:500, GB12105, Servicebio, China), anti‐GFAP rabbit pAb (1:1000, GB111096, Servicebio, China), anti‐phospho‐AMPK alpha 1 (T183) + AMPK alpha 2 (T172) rabbit pAb (1:2000, GB114323, Servicebio, China), and anti‐PGC1 alpha+beta rabbit pAb (1:2000, GB11912, Servicebio, China). Secondary antibodies were applied and incubated with brain sections for 1 h at 25 °C, including Cy3 conjugated goat anti‐rabbit IgG (H+L) (1:300, GB21303, Servicebio, China), Cy3 conjugated goat anti‐mouse IgG (H+L) (1:300, GB21301, Servicebio, China), Alexa Fluor 488‐conjugated conjugated goat anti‐rabbit IgG (H+L) (1:400, GB25303, Servicebio, China), and HRP conjugated goat anti‐rabbit IgG (H+L) (1:500, GB23303, Servicebio, China). Double fluorescence staining of primary antibodies from the same species was achieved by using tyramide signal amplification (TSA) reagents corresponding to different fluorescent wavelengths (iF488‐Tyramide, G1231, 1:500, Servicebio, China; iF555‐Tyramide, G1233, 1:500, Servicebio, China). Micrographs were taken using a digital section scanner (Pannoramic MIDI, 3DHISTECH Ltd., Hungary), and quantitative data on the average fluorescence intensity of these micrographs were calculated using ImageJ software.

### Western Blot (WB) and Superoxide Dismutase (SOD) Activity Analyses for Brain Tissue

Extracted brain tissue was homogenized in a modified radioimmunoprecipitation buffer supplemented with a 10 µL mL^−1^ protease inhibitor mixture. The extracts were then centrifuged at 12000 rpm and the supernatant was gathered for protein determination. After quantifying the protein concentration, the subsequent experimental steps, secondary antibodies, and partial primary antibodies were the same as described in Section “Preparation, Physicochemical Properties, and Rheological Analysis of OACDP Hydrogels” in Experimental Section for the in vitro cellular WB experiments, containing anti‐GAPDH antibody (≈37 kDa), anti‐GFAP antibody (≈55 kDa), HRP‐labeled goat anti‐rabbit IgG(H+L), and HRP‐labeled goat anti‐mouse IgG(H+L). Additional primary antibodies used for tissue WB analysis were included: anti‐tyrosine hydroxylase antibody (ab137869, Abcam, UK, 1:5000, ≈58 kDa), PGC1 alpha antibody (AF8600, Affinity, China, 1:1000, ≈91 kDa), Iba1 antibody (sc‐32725, Santa Cruz, USA, 1:500, ≈21 kDa), AMPK alpha antibody (AF6423, Affinity, China, 1:1000, ≈62 kDa), phospho‐AMPK alpha antibody (AF3423, Affinity, China, 1:1000, ≈62 kDa). The SOD activity of the targeted brain region was determined by a Total Superoxide Dismutase Assay Kit with WST‐8 (Beyotime, China) and was operated according to the manufacturer's instructions. The measurement with the SOD activity assay was collected at 450 nm by the microplate reader.

### Statistical Analysis

At least three replications were performed to eliminate unexpected situations. All quantitative results were obtained by independent experiments. Calculated data were shown as mean ± standard deviation. One‐way ANOVA followed by Tukey's post‐hoc test was utilized to statistically differentiate between groups. Two‐way ANOVA with Tukey's post‐hoc test was employed to analyze the behavioral data. Behavioral tests were conducted at multiple time points on the same animal and were analyzed with a two‐way ANOVA using a repeated measures design, i.e., time factor. The computations were carried out using the commercially available GraphPad Prism 9 software. A calculated *p*‐value of less than 0.05 was considered statistically significant between the two groups. The statistical results of the one‐way ANOVA and two‐way ANOVA that appeared in the manuscript, containing F values with degrees of freedom and *p* values, are summarized in tables S1, S2 (Supporting Information), respectively.

## Conflict of Interest

The authors declare no conflict of interest.

## Supporting information



Supporting Information

## Data Availability

The data that support the findings of this study are available from the corresponding author upon reasonable request.

## References

[1] a) A. Toulouse , A. M. Sullivan , Prog. Neurobiol. 2008, 85, 376; 18582530 10.1016/j.pneurobio.2008.05.003

[2] a) M. C. Rodriguez‐Oroz , M. Rodriguez , J. Guridi , K. Mewes , V. Chockkman , J. Vitek , M. R. DeLong , J. A. Obeso , Brain 2001, 124, 1777; 11522580 10.1093/brain/124.9.1777

[3] E. Kip , L. C. Parr‐Brownlie , Ageing Res. Rev. 2022, 78, 101618. 35395416 10.1016/j.arr.2022.101618

[4] a) C. Marogianni , M. Sokratous , E. Dardiotis , G. M. Hadjigeorgiou , D. Bogdanos , G. Xiromerisiou , Int. J. Mol. Sci. 2020, 21, 8421; 33182554 10.3390/ijms21228421 PMC7697354

[5] K. McFarthing , G. Rafaloff , M. Baptista , L. Mursaleen , R. Fuest , R. K. Wyse , S. R. W. Stott , J. Parkinson's Dis. 2022, 12, 1073. 35527571 10.3233/JPD-229002 PMC9198738

[6] a) E. Tolosa , A. Garrido , S. W. Scholz , W. Poewe , Lancet Neurol. 2021, 20, 385; 33894193 10.1016/S1474-4422(21)00030-2 PMC8185633

[7] a) M. J. Armstrong , M. S. Okun , JAMA, J. Am. Med. Assoc. 2020, 323, 548; 10.1001/jama.2019.22360 32044947

[8] S. D. Aradi , R. A. Hauser , Neurotherapeutics 2020, 17, 1339. 32761324 10.1007/s13311-020-00889-4 PMC7851275

[9] S. A. Schneider , R. N. Alcalay , J. Neurol. 2020, 267, 860. 31974807 10.1007/s00415-020-09705-7 PMC7035220

[10] a) J. Li , D. J. Mooney , Nat. Rev. Mater. 2016, 1, 16071; 29657852 10.1038/natrevmats.2016.71 PMC5898614

[11] J. Xu , S.‐h. Hsu , J. Biomed. Sci. 2023, 30, 43. 37340481 10.1186/s12929-023-00939-x PMC10280980

[12] Y. Tu , N. Chen , C. Li , H. Liu , R. Zhu , S. Chen , Q. Xiao , J. Liu , S. Ramakrishna , L. He , Acta Biomater. 2019, 90, 1. 30951899 10.1016/j.actbio.2019.03.057

[13] M. M. H. Rumon , A. A. Akib , F. Sultana , M. Moniruzzaman , M. S. Niloy , M. S. Shakil , C. K. Roy , Polymers 2022, 14, 4539. 36365532 10.3390/polym14214539 PMC9654449

[14] J. Zhang , Y. Fu , P. Yang , X. Liu , Y. Li , Z. Gu , Adv. Mater. Interfaces 2020, 7, 2000632.

[15] J. Xu , T.‐Y. Chen , C.‐H. Tai , S.‐h. Hsu , Biomater. Res. 2023, 27, 8. 36755333 10.1186/s40824-023-00347-0 PMC9909866

[16] Y. Liu , J. Deng , Y. Liu , W. Li , X. Nie , Front. Pharmacol. 2021, 12, 675725. 34234672 10.3389/fphar.2021.675725 PMC8255968

[17] X. Li , Fibroblast Growth Factors, Elsevier, Amsterdam, The Netherland 2023.

[18] a) C. Rühlmann , D. Dannehl , M. Brodtrück , A. C. Adams , J. Stenzel , T. Lindner , B. J. Krause , B. Vollmar , A. Kuhla , J. Alzheimer's Dis. 2021, 80, 357; 33554901 10.3233/JAD-200837

[19] a) C. Yang , W. Wang , P. Deng , C. Li , L. Zhao , H. Gao , Front. Aging Neurosci. 2021, 13, 778527; 35002679 10.3389/fnagi.2021.778527 PMC8727910

[20] a) S.‐H. Lin , C. M. Papadakis , J.‐J. Kang , J.‐M. Lin , S.‐h. Hsu , Chem. Mater. 2021, 33, 3945;

[21] a) K. Saha , A. J. Keung , E. F. Irwin , Y. Li , L. Little , D. V. Schaffer , K. E. Healy , Biophys. J. 2008, 95, 4426; 18658232 10.1529/biophysj.108.132217 PMC2567955

[22] a) L. Lu , S. J. Peter , M. D. Lyman , H.‐L. Lai , S. M. Leite , J. A. Tamada , S. Uyama , J. P. Vacanti , L. Robert , A. G. Mikos , Biomaterials 2000, 21, 1837; 10919687 10.1016/s0142-9612(00)00047-8

[23] F. A. Khan , A. Maalik , G. Murtaza , J. Food Drug Anal. 2016, 24, 695. 28911606 10.1016/j.jfda.2016.05.003 PMC9337298

[24] Y. Ren , B. Wei , X. Song , N. An , Y. Zhou , X. Jin , Y. Zhang , Int. J. Neurosci. 2015, 125, 555. 25171224 10.3109/00207454.2014.959121

[25] Y. Fu , W. J. Kao , Expert Opin. Drug Deliv. 2010, 7, 429. 20331353 10.1517/17425241003602259 PMC2846103

[26] S. Dash , P. N. Murthy , L. Nath , P. Chowdhury , Acta Pol. Pharm. 2010, 67, 217. 20524422

[27] T. Yamamoto , S. Yuki , T. Watanabe , M. Mitsuka , K.‐I. Saito , K. Kogure , Brain Res. 1997, 762, 240. 9262182 10.1016/s0006-8993(97)00490-3

[28] S. J. Cha , K. Kim , Antioxidants 2022, 11, 195. 35204078 10.3390/antiox11020195 PMC8868074

[29] Y. Pan , W. Li , Y. Feng , J. Xu , H. Cao , Exp. Ther. Med. 2020, 19, 1407. 32010316 10.3892/etm.2019.8351 PMC6966113

[30] Y.‐C. E. Li , Y. A. Jodat , R. Samanipour , G. Zorzi , K. Zhu , M. Hirano , K. Chang , A. Arnaout , S. Hassan , N. Matharu , A. Khademhosseini , M. Hoorfar , S. R. Shin , Biofabrication 2021, 13, 015014. 10.1088/1758-5090/abc1be PMC8387028 33059333

[31] G. Bonvento , J. P. Bolaños , Cell Metab. 2021, 33, 1546. 34348099 10.1016/j.cmet.2021.07.006

[32] S. Bhatt , L. Puli , C. R. Patil , Drug Discov. Today 2021, 26, 794. 33306995 10.1016/j.drudis.2020.12.004

[33] a) T. H. Romeiro , S. C. Da Silva , P. d. S. Beggiora , G. B. Sampaio , R. A. Brandão , M. V. Santos , H. R. Machado , L. d. S. Lopes , J. Chem. Neuroanat. 2022, 119, 102059; 34896559 10.1016/j.jchemneu.2021.102059

[34] a) F. Blandini , G. Levandis , E. Bazzini , G. Nappi , M. T. Armentero , Eur. J. Neurosci. 2007, 25, 397; 17284180 10.1111/j.1460-9568.2006.05285.x

[35] X. Fang , J. Ma , D. Mu , B. Li , B. Lian , C. Sun , Neurotox. Res. 2020, 37, 616. 31997152 10.1007/s12640-019-00151-6

[36] K. Guo , W. Huang , K. Chen , P. Huang , W. Peng , R. Shi , T. He , M. Zhang , H. Wang , J. Hu , X. Wang , Y. Shentu , H. Xu , L. Lin , Aging Cell 2023, 22, e13937. 37503695 10.1111/acel.13937 PMC10497839

[37] a) J. J. Chen , L. Marsh , Ther. Adv. Neurolog. Disord. 2013, 7, 52; 10.1177/1756285613495723 PMC3886380 24409202

[38] I. Deng , S. Garg , X.‐F. Zhou , L. Bobrovskaya , Front. Biosci. 2022, 14, 13. 10.31083/j.fbs1402013 35730438

[39] a) N. Usui , M. Yoshida , Y. Takayanagi , N. Nasanbuyan , A. Inutsuka , H. Kurosu , H. Mizukami , Y. Mori , M. Kuro‐o , T. Onaka , J. Neuroendocrinol. 2021, 33, e13026; 34472154 10.1111/jne.13026 PMC9285091

[40] a) T. T. Lai , B. Gericke , M. Feja , M. Conoscenti , M. Zelikowsky , F. Richter , npj Parkinson's Dis. 2023, 9, 97; 37349373 10.1038/s41531-023-00547-4 PMC10287685

[41] a) J. L. Hudson , C. G. van Horne , I. Strömberg , S. Brock , J. Clayton , J. Masserano , B. J. Hoffer , G. A. Gerhardt , Brain Res. 1993, 626, 167; 8281427 10.1016/0006-8993(93)90576-9

[42] a) A. Singh , P. Tripathi , A. K. Yadawa , S. Singh , Neurochem. Res. 2020, 45, 1731; 32462543 10.1007/s11064-020-03058-3

[43] N. Xiong , J. Xiong , G. Khare , C. Chen , J. Huang , Y. Zhao , Z. Zhang , X. Qiao , Y. Feng , H. Reesaul , Y. Zhang , S. Sun , Z. Lin , T. Wang , PLoS One 2011, 6, e20677. 21677777 10.1371/journal.pone.0020677 PMC3108992

[44] a) N. Simola , M. Morelli , A. R. Carta , Neurotox. Res. 2007, 11, 151; 17449457 10.1007/BF03033565

[45] E. C. Hirsch , T. Breidert , E. Rousselet , S. Hunot , A. Hartmann , P. P. Michel , Ann. N. Y. Acad. Sci. 2003, 991, 214. 12846989 10.1111/j.1749-6632.2003.tb07478.x

[46] a) S. A. Zaitone , E. Ahmed , N. M. Elsherbiny , E. T. Mehanna , M. K. El‐Kherbetawy , M. H. ElSayed , D. M. Alshareef , Y. M. Moustafa , Pharmacol. Rep. 2019, 71, 32; 30368226 10.1016/j.pharep.2018.08.004

[47] a) J. Li , M. Darabi , J. Gu , J. Shi , J. Xue , L. Huang , Y. Liu , L. Zhang , N. Liu , W. Zhong , L. Zhang , M. Xing , L. Zhang , Biomaterials 2016, 102, 72; 27322960 10.1016/j.biomaterials.2016.06.016

[48] K. Kikuchi , S. Tancharoen , N. Takeshige , M. Yoshitomi , M. Morioka , Y. Murai , E. Tanaka , Int. J. Mol. Sci. 2013, 14, 13909. 23880849 10.3390/ijms140713909 PMC3742225

[49] S. Matsumoto , M. Murozono , M. Kanazawa , T. Nara , T. Ozawa , Y. Watanabe , Acute Med. Surg. 2018, 5, 213. 29988669 10.1002/ams2.343 PMC6028804

[50] a) M. Balteau , A. Van Steenbergen , A. D. Timmermans , C. Dessy , G. Behets‐Wydemans , N. Tajeddine , D. Castanares‐Zapatero , P. Gilon , J.‐L. Vanoverschelde , S. Horman , L. Hue , L. Bertrand , C. Beauloye , Am. J. Physiol.: Heart Circ. Physiol. 2014, 307, H1120; 25128166 10.1152/ajpheart.00210.2014

[51] X. Zhang , J. K. Jackson , H. M. Burt , Int. J. Pharm. 1996, 132, 195.

[52] a) S. Zhu , Y. Ying , Q. Wu , Z. Ni , Z. Huang , P. Cai , Y. Tu , W. Ying , J. Ye , R. Zhang , Y. Zhang , M. Chen , Z. Xiang , H. Dou , Q. Huang , X. Li , H. He , J. Xiao , Q. Ye , Z. Wang , Chem. Eng. J. 2021, 426, 130827;

[53] A. Benazzouz , D. M. Gao , Z. G. Ni , B. Piallat , R. Bouali‐Benazzouz , A.‐L. Benabid , Neuroscience 2000, 99, 289. 10938434 10.1016/s0306-4522(00)00199-8

[54] G. Paxinos , C. R. Watson , P. C. Emson , J. Neurosci. Methods 1980, 3, 129. 6110810 10.1016/0165-0270(80)90021-7

[55] C.‐F. Su , L. Jiang , X.‐W. Zhang , A. Iyaswamy , M. Li , Front. Pharmacol. 2021, 12, 644219. 33967780 10.3389/fphar.2021.644219 PMC8100515

[56] X. Fang , X. Zhou , Y. Miao , Y. Han , J. Wei , T. Chen , AMB Express 2020, 10, 80. 32333225 10.1186/s13568-020-01014-6 PMC7182653

