# Supplementary material for: Injectable Hierarchical Bioactive Hydrogels with Fibroblast Growth Factor 21/Edaravone/Caffeic Acid Asynchronous Delivery for Treating Parkinson's Disease
Source: Adv Sci (Weinh). 2024 Dec 4;12(4):2412020. doi: 10.1002/advs.202412020 (PMC11775539; doi:10.1002/advs.202412020)
Supplement: Supplementary file 1 — Supporting Information [file ADVS-12-2412020-s001.docx]

**Supporting information**

**Injectable hierarchical bioactive hydrogels with triple-drug asynchronous delivery for treating Parkinson's disease**

Junpeng Xu ^1, 2, 3, 4, †^, Peng Dai ^2, 3, 4, †^, Chen Zhang ^5, †^, Na Dong ^2, 3, 4^, Caiyan Li ^2, 3, 4^, Chonghui Tang ^1^, Zhihao Jin ^2, 3, 4^, Shih-Ho Lin ^6^, Luyang Ye ^2, 3, 4^, Tianmiao Sun ^1^, Yukai Jin ^1^, Fenzan Wu ^1^, Lihua Luo ^5^, Ping Wu ^2, 3, 4^, Shengcun Li ^2, 3, 7^, Xiaokun Li ^2, 3, 4, *^, Shan-hui Hsu ^6, 8, *^, Dawei Jiang ^1, 2, 3, 4, *^, Zhouguang Wang ^2, 3, 4, *^

^1^ Affiliated Cixi Hospital, Wenzhou Medical University, Ningbo 315300, Zhejiang, China

^2^ School of Pharmaceutical Science, Wenzhou Medical University, Wenzhou 325035, Zhejiang, China

^3^ Oujiang Laboratory (Zhejiang Lab for Regenerative Medicine, Vision and Brain Health), School of Pharmaceutical Science, Wenzhou Medical University, Wenzhou 325035, Zhejiang, China

^4^ National Key Laboratory of Macromolecular Drug Development and Manufacturing, School of Pharmaceutical Science, Wenzhou Medical University, Wenzhou 325035, Zhejiang, China

^5^ School and Hospital of Stomatology, Wenzhou Medical University, Wenzhou 324025, Zhejiang, China

^6^ Institute of Polymer Science and Engineering, National Taiwan University, Taipei 106319, Taiwan, Republic of China

^7^ Rehabilitation Medicine Center, The Second Affiliated Hospital and Yuying Children’s Hospital of Wenzhou Medical University, Wenzhou 325000, Zhejiang, China

^8^ Institute of Cellular and System Medicine, National Health Research Institutes, Miaoli 350401, Taiwan, Republic of China

^†^ These authors contributed equally to this paper

**Supporting Table: 3**

**Supporting Figures: 15**

***Corresponding authors:**

**Prof. Dr. Zhouguang Wang:** Email: [wangzhouguang@wmu.edu.cn](mailto:wangzhouguang@wmu.edu.cn)

**Dr. Dawei Jiang:** Email: [jiangdawei@wmu.edu.cn](mailto:jiangdawei@wmu.edu.cn)

**Prof. Dr. Shan-hui Hsu:** Email: [shhsu@ntu.edu.tw](mailto:shhsu@ntu.edu.tw)

**Prof. Dr. Xiaokun Li:** Email: [xiaokunli@wmu.edu.cn](mailto:xiaokunli@wmu.edu.cn)

**Table S1.** The summarized statistical results of one-way ANOVA in the whole study.

| **Figure No.** | **F (degree of freedom)** | | **P value** |
| --- | --- | --- | --- |
|  | [DF_between-group_, DF_within-group_] | Value |  |
| Figure 2H | [5, 12] | 132.5 | P<0.0001 |
| Figure S6 | [4, 10] | 2.882 | P=0.0795 |
| Figure S7 | [4, 10] | 1.657 | P=0.2355 |
| Figure S8 | [5, 12] | 564.9 | P<0.0001 |
| Figure 4D | [3, 8] | 293.3 | P<0.0001 |
| Figure 4F | [4, 15] | 9.520 | P=0.0005 |
| Figure 4G | [4, 15] | 22.67 | P<0.0001 |
| Figure 4H | [4, 15] | 26.02 | P<0.0001 |
| Figure 5E | [5, 18] | 397.9 | P<0.0001 |
| Figure 5F | [5, 18] | 77.88 | P<0.0001 |
| Figure 5G | [5, 18] | 53.36 | P<0.0001 |
| Figure 6C | [5, 18] | 696.1 | P<0.0001 |
| Figure S11B | [5, 18] | 1022 | P<0.0001 |
| Figure 6E | [5, 18] | 500.4 | P<0.0001 |
| Figure 6G | [5, 12] | 114.6 | P<0.0001 |
| Figure 6H | [5, 12] | 76.66 | P<0.0001 |
| Figure S13 | [5, 18] | 73.2 | P<0.0001 |
| Figure 7B | [5, 18] | 1292 | P<0.0001 |
| Figure 7C | [5, 18] | 1108 | P<0.0001 |
| Figure 7E | [5, 12] | 84.62 | P<0.0001 |
| Figure 7F | [5, 12] | 88.72 | P<0.0001 |
| Figure 7H | [5, 18] | 1714 | P<0.0001 |
| Figure 7I | [5, 18] | 968.3 | P<0.0001 |
| Figure 7K | [5, 12] | 309.1 | P<0.0001 |
| Figure 7L | [5, 12] | 243.4 | P<0.0001 |

**Table S2.** The summarized statistical results of two-way ANOVA in the whole study.

| **Figure No.** |  | **F (degree of freedom)** | | **P value** |
| --- | --- | --- | --- | --- |
|  |  | [DF_between-group_, DF_within-group_] | Value |  |
| Figure 5B | Time × Groups | [8, 20] | 16.67 | P<0.0001 |
|  | Time | [1.504, 15.04] | 113.7 | P<0.0001 |
|  | Groups | [4, 10] | 41.59 | P<0.0001 |
| Figure 5C | Time × Groups | [8, 20] | 33.73 | P<0.0001 |
|  | Time | [1.110, 11.10] | 0.6872 | P=0.4396 |
|  | Groups | [4, 10] | 191.6 | P<0.0001 |

**Table S3.** Basic properties of the hydrogels with different formulae.

| **Name** | **pOAC (wt%)** | **DPX (wt%)** | **Self-healing time (min)** | **Porosity (%)** | **Swelling ratio (%)** | **Storage modulus (Pa)** |
| --- | --- | --- | --- | --- | --- | --- |
| OACDP1 | 1 | 5 | ~1 | 96.4 ± 2.4 | Untestable | ~8 |
| OACDP2 | 2 | 5 | ~1 | 95.7 ± 2.9 | Untestable | ~14 |
| OACDP3 | 3 | 5 | ~2 | 93.4 ± 3.1 | Untestable | ~45 |
| OACDP4 | 4 | 5 | ~6 | 92.7 ± 1.7 | 148.1 ± 24.7 | ~72 |
| OACDP | 5 | 5 | ~10 | 90.1 ± 2.2 | 115.4 ± 10.3 | ~167 |





**Figure S1.** ^1^H NMR spectrum of poly(OEGMA-*c*-AEMA).


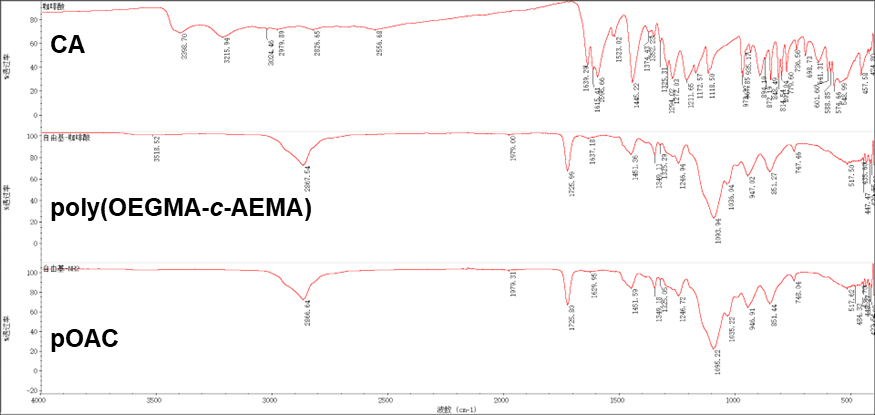


**Figure S2.** FTIR spectra of CA, poly(OEGMA-*c*-AEMA), and pOAC.


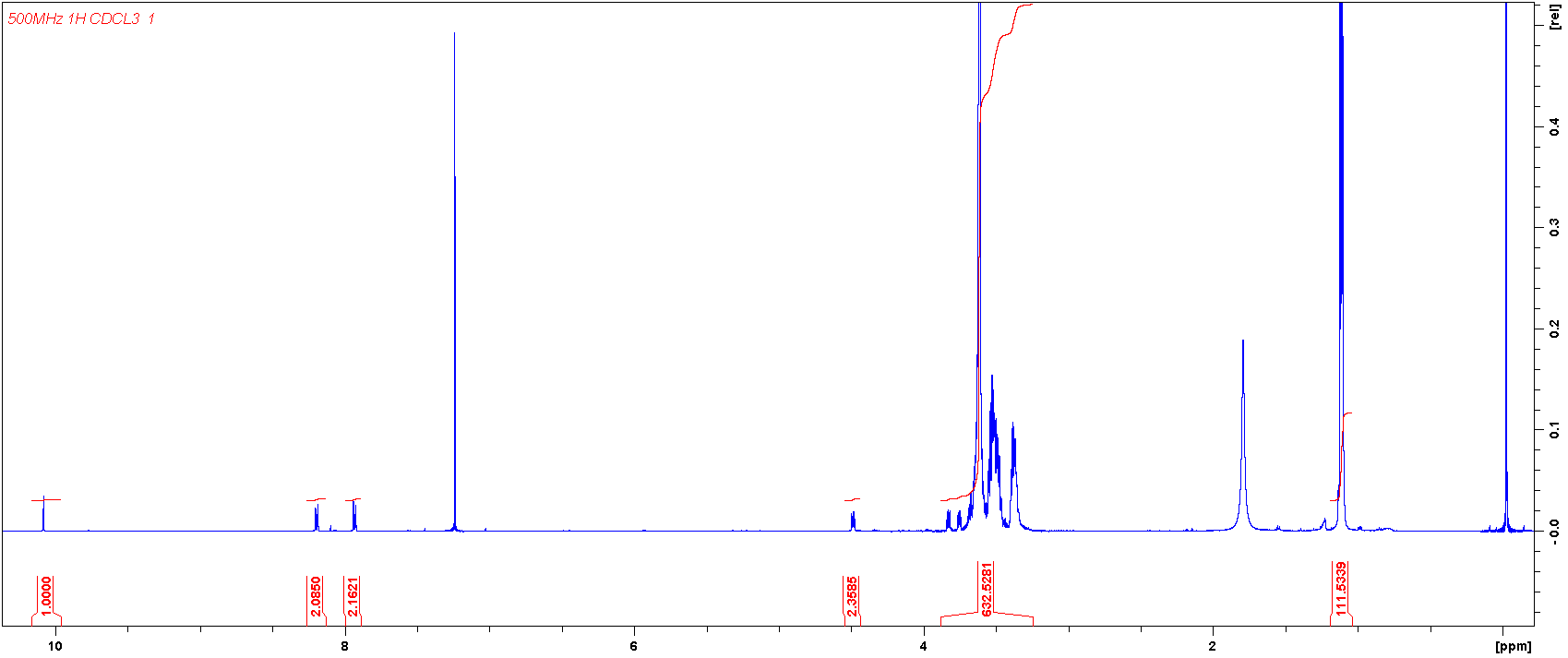


**Figure S3.** The ^1^H NMR spectrum of DPX.


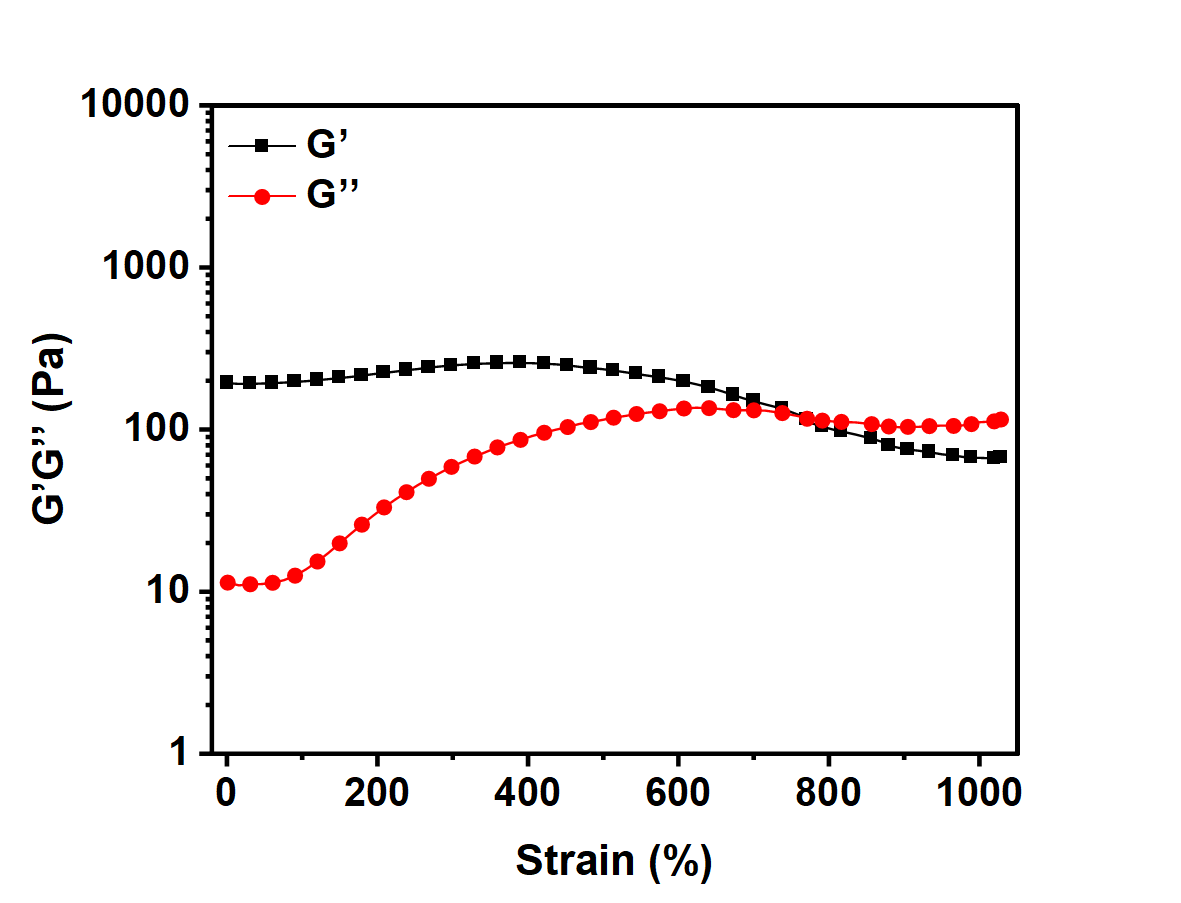


**~760%**

**Figure S4.** Rheological data by strain sweep experiments of the OACDP hydrogel in the range of 0.1 to 800% dynamic strain amplitudes at 1 Hz frequency. Black arrows show the gel-to-sol points and the corresponding strain values.


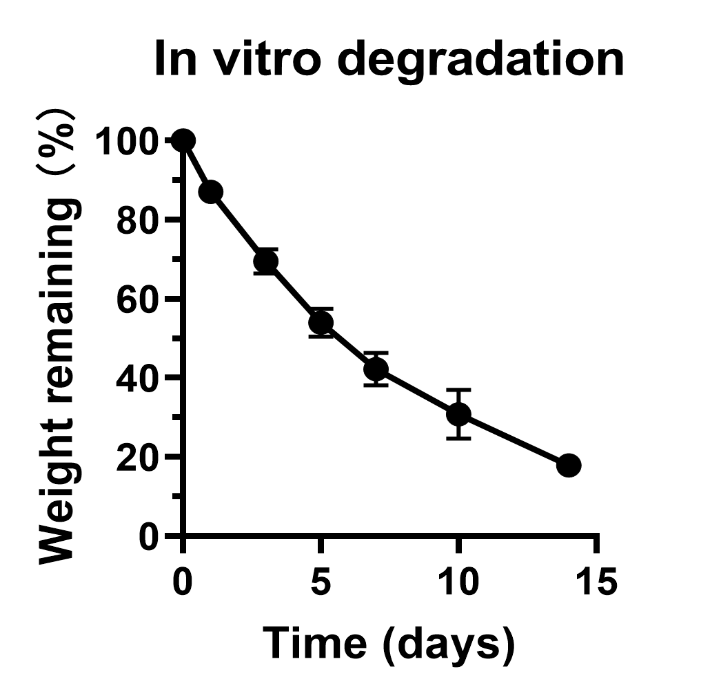


**Figure S5.** Remaining weights of the OACDP hydrogel in PBS at 37 °C. Data are represented as mean ± SD (n ≥ 3).


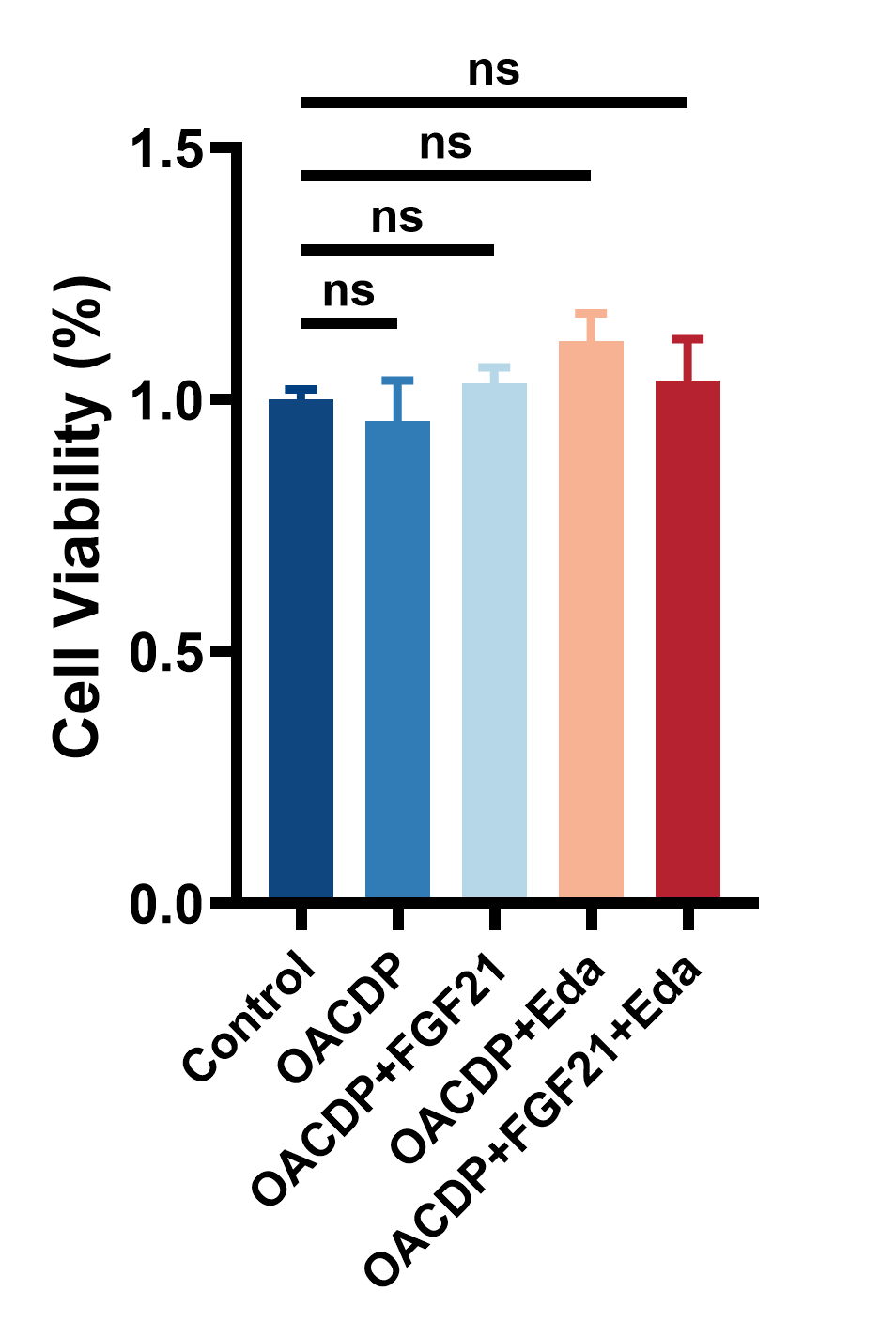


**Figure S6.** Cell viability of NE-4C NSCs after co-incubation with various hydrogels using Transwell chambers for 24 h. All data were normalized to the control group (without any treatment). Data are represented as mean ± SD (n ≥ 3).


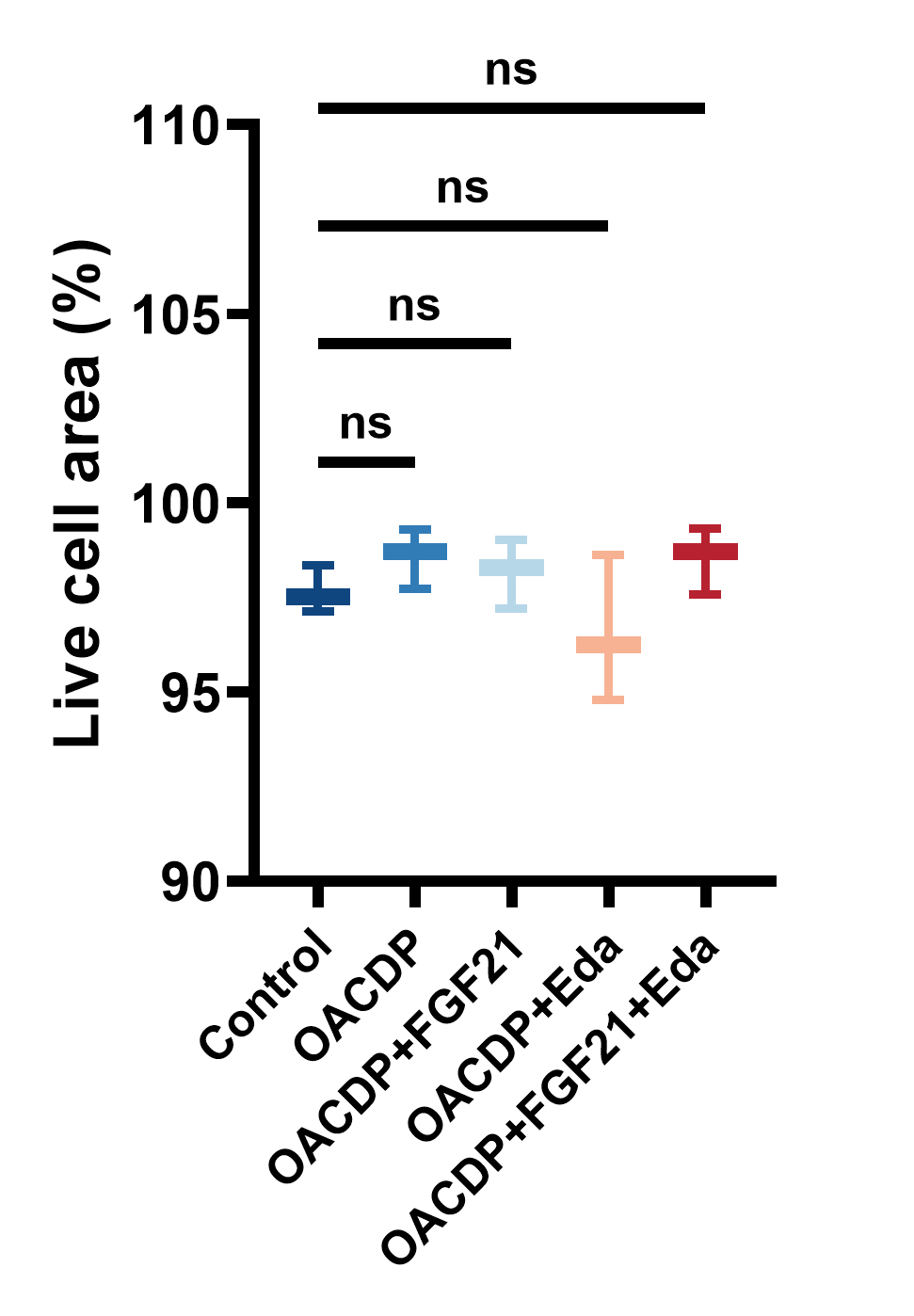


**Figure S7.** Quantification of live/dead NSCs after a 5-day culture period. Data are represented as mean ± SD (n ≥ 3).


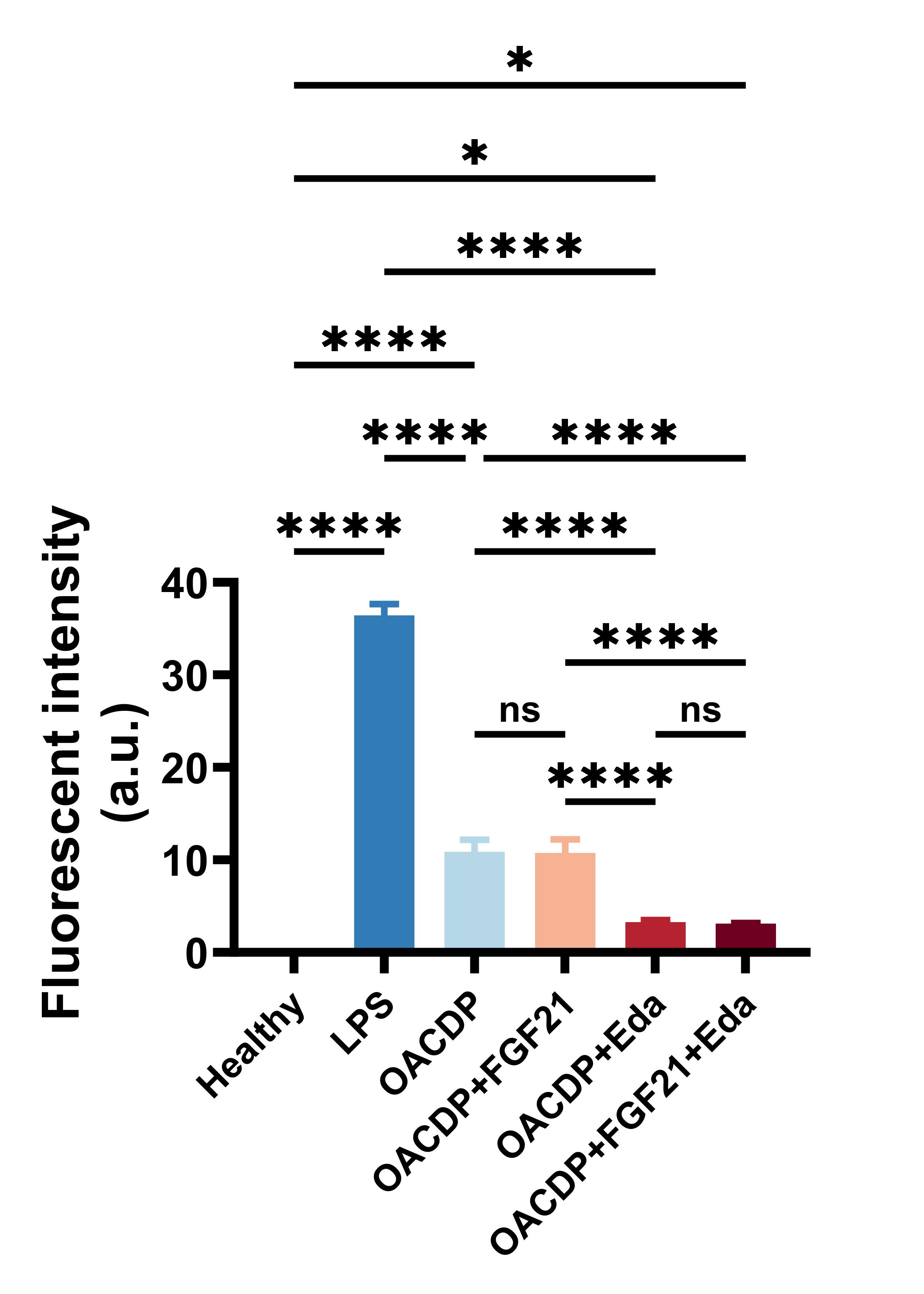


**Figure S8.** Quantification of ROS assay after LPS-induced NSCs. Data are represented as mean ± SD (n ≥ 3). **p* < 0.05, ***p* < 0.01, ****p* < 0.001, and *****p* < 0.0001 between the indicated groups.


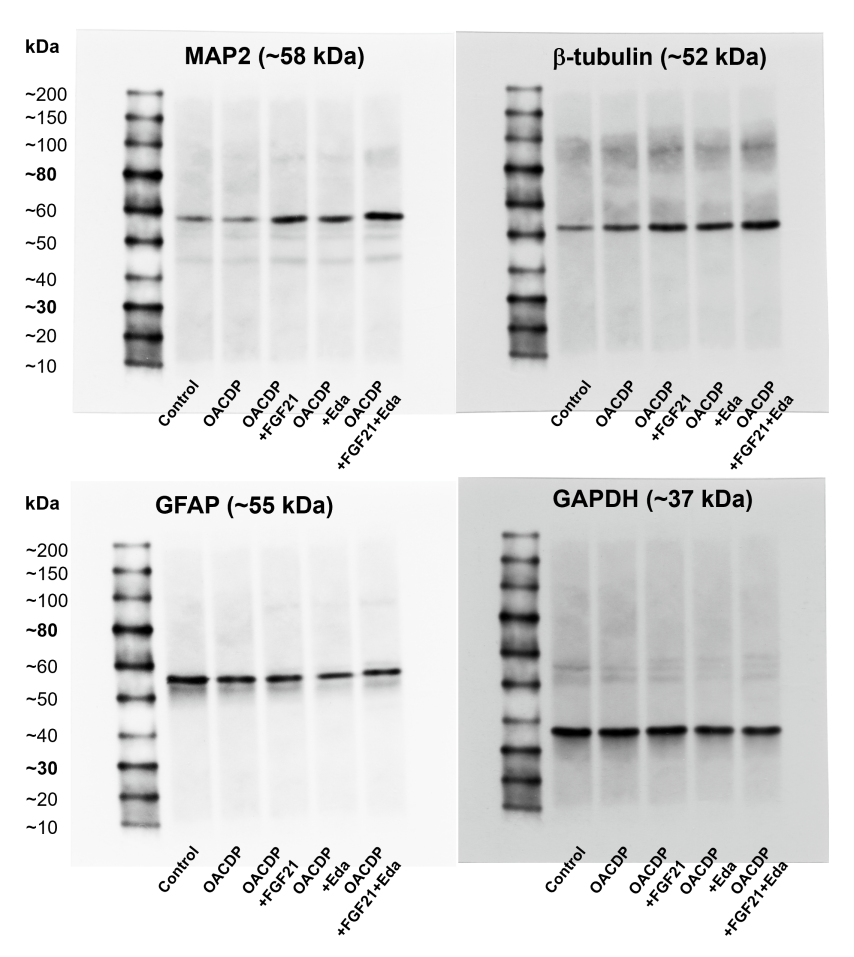


**Figure S9.** The uncropped raw images of WB for GAPDH, GFAP, MAP2, and β-tubulin in cell experiments.


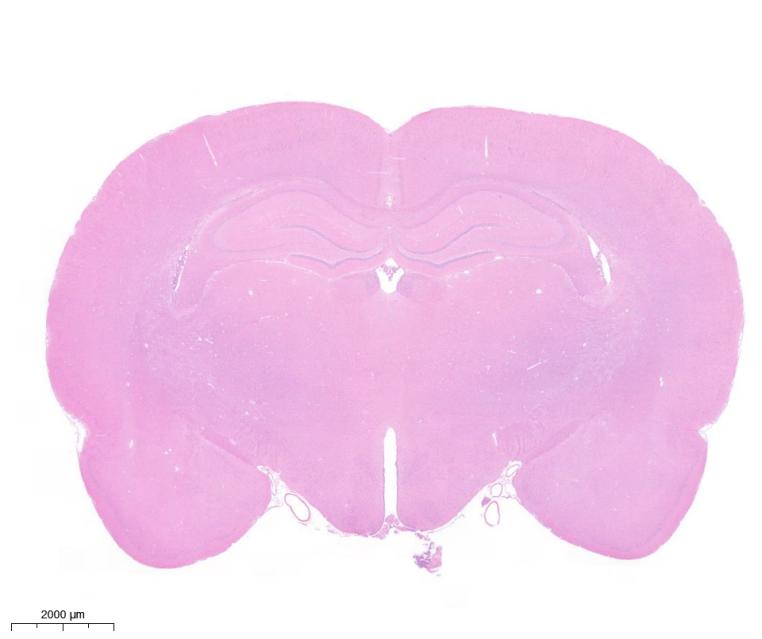


**Figure S10.** The H&E staining for the OACDP-injected PD rat after 14 days of treatment, indicating no hydrogel residue.


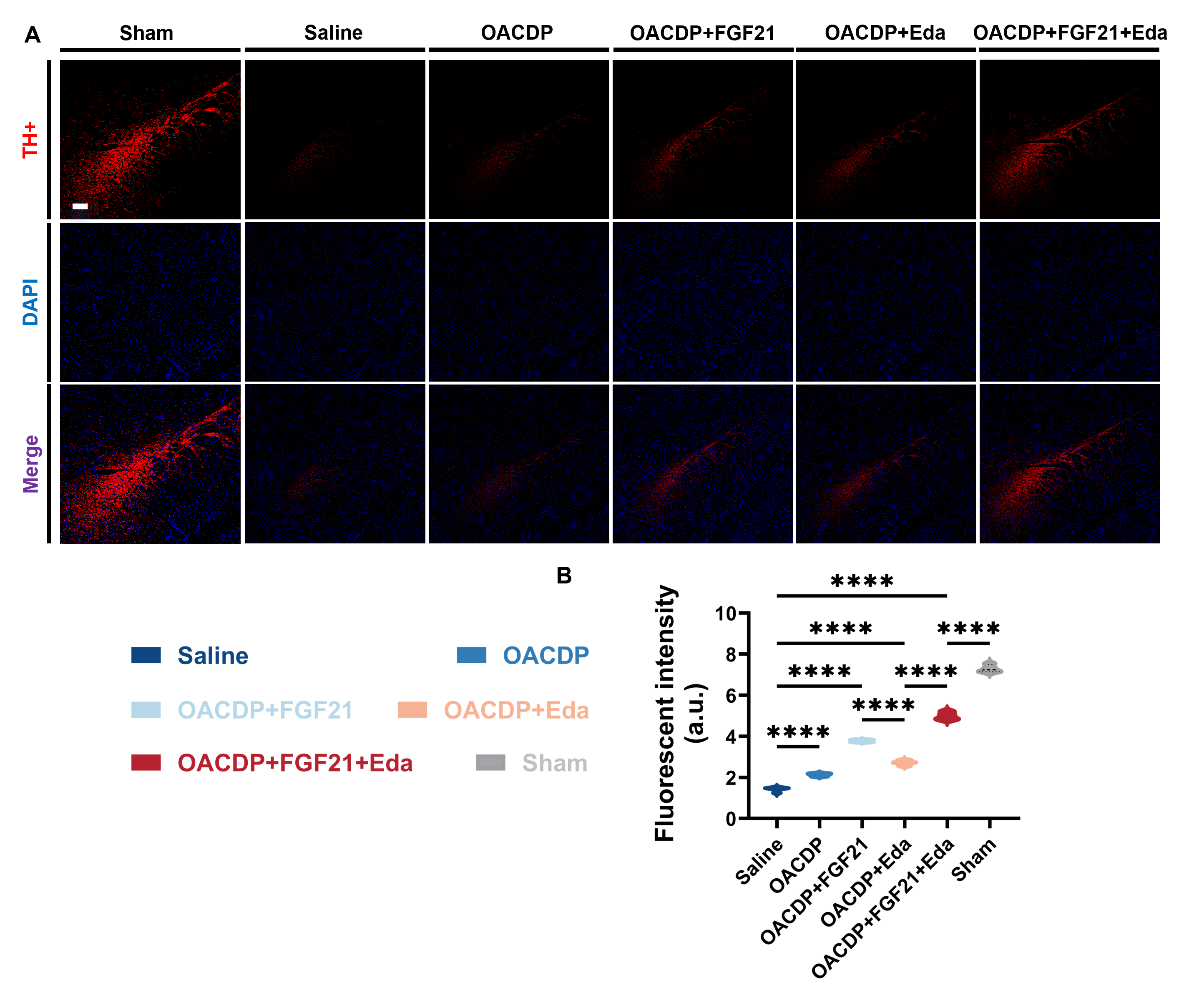


**Figure S11.** (A) The expression of TH+ dopaminergic fibers in SNc was investigated and validated at 14 days after the second surgery injection. (B) The average fluorescent intensities were quantified and presented as graphics for TH+ dopaminergic fibers in SNc. The scale bars represent for 200 μm. Data are represented as mean ± SD (n ≥ 3). *****p* < 0.0001 between the indicated groups.


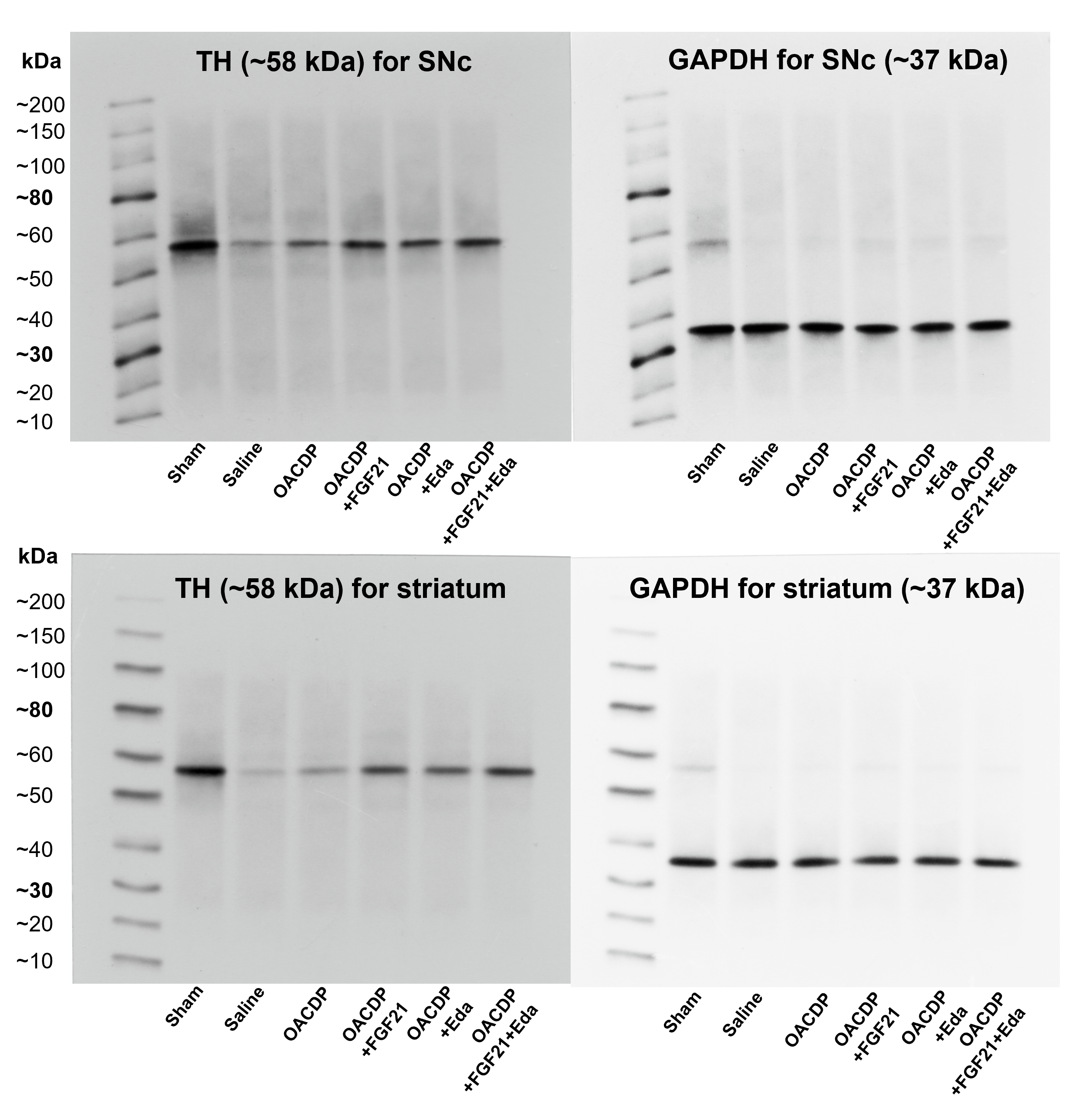


**Figure S12.** The uncropped raw images of WB analysis for TH in two brain regions, including SNc and striatum.


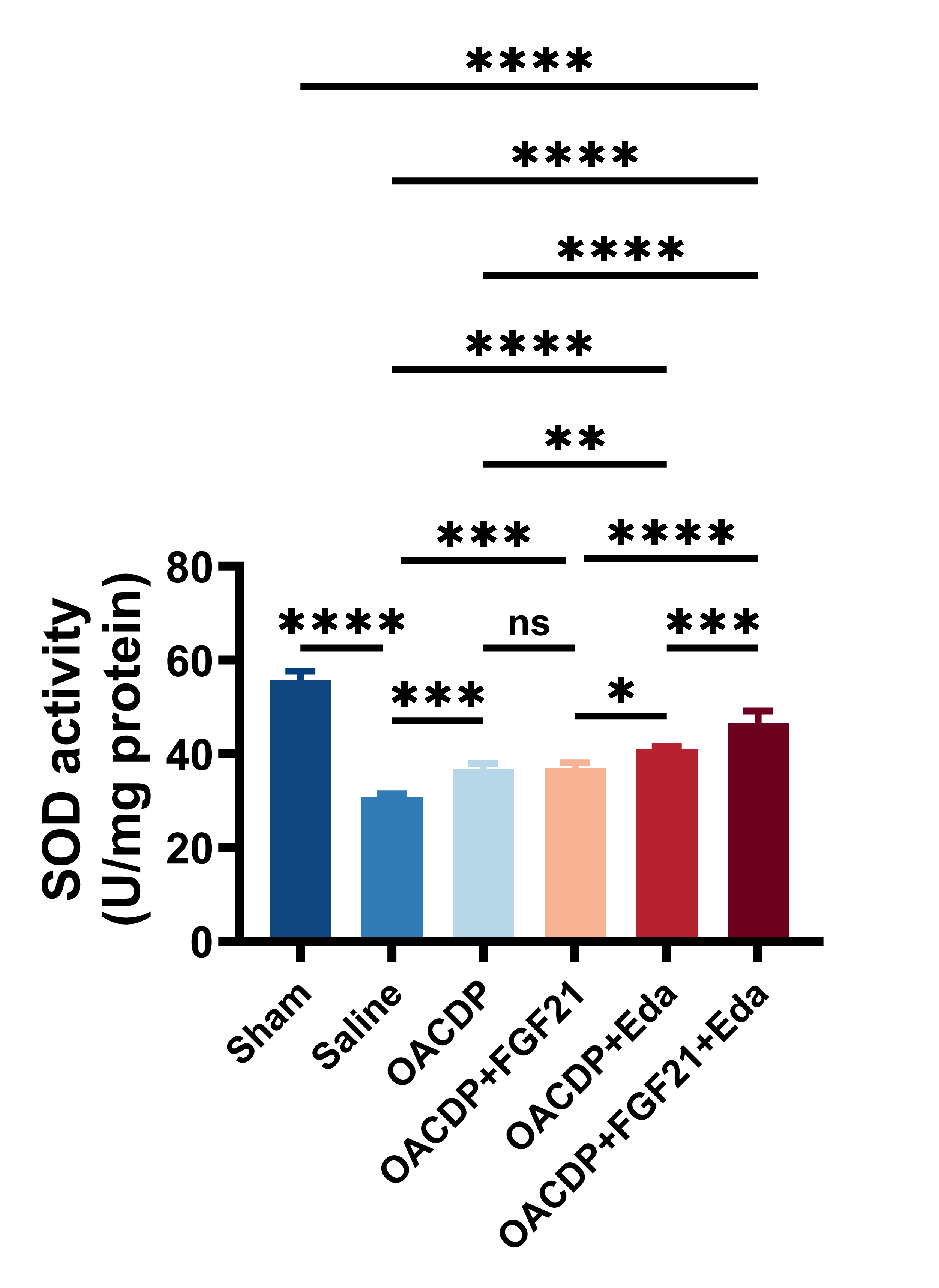


**Figure S13.** Quantification of total SOD activity assay for each group after treatment. Data are represented as mean ± SD (n ≥ 3). **p* < 0.05, ***p* < 0.01, ****p* < 0.001, and *****p* < 0.0001 between the indicated groups.


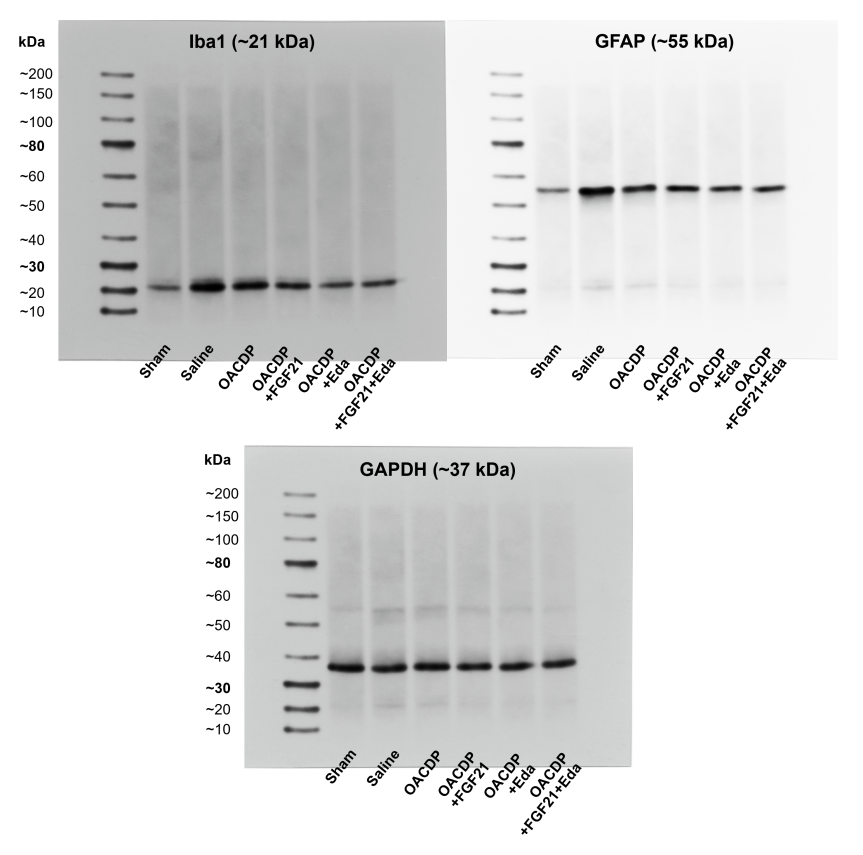


**Figure S14.** The uncropped raw images of WB analysis including Iba1, GFAP, and GAPDH for brain tissue.


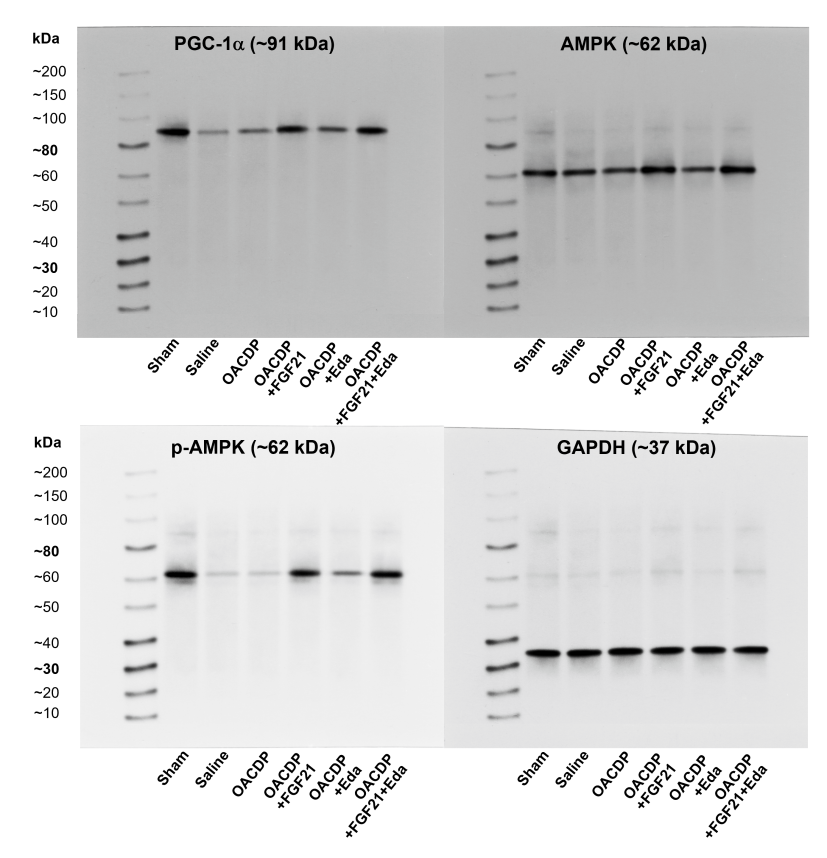


**Figure S15.** The uncropped raw images of WB analysis including AMPK, p-AMPK, PGC-1α, and GAPDH for brain tissue.
